# Integrated Global Phosphoproteomic, Bioinformatic, and Machine Learning Analysis Reveals Regulatory Networks of TOP1, TOP2A, TOP2B, and C1orf35 in Hepatocellular Carcinoma (HCC)

**DOI:** 10.32604/or.2026.073745

**Published:** 2026-04-22

**Authors:** Aktham Mestareehi

**Affiliations:** 1 Department of Applied Pharmaceutical Sciences and Clinical Pharmacy, Faculty of Pharmacy, Isra University, Amman, Jordan; 2 Department of Pharmaceutical Sciences, Eugene Applebaum College of Pharmacy and Health Sciences, Wayne State University, Detroit, MI, USA

**Keywords:** Hepatocellular carcinoma (HCC), phosphoproteomics, proteomics, bioinformatics, the Kaplan-Meier method, nano–liquid chromatography coupled with electrospray ionization tandem mass spectrometry (nLC-ESI-MS/MS), c-BioPortal

## Abstract

**Objective:**

Hepatocellular carcinoma (HCC) is a leading cause of cancer-related mortality worldwide, largely due to late diagnosis, molecular heterogeneity, and limited prognostic biomarkers. Aberrant protein phosphorylation plays a critical role in cancer progression by regulating DNA damage response, cell cycle control, and signaling pathways; however, the prognostic relevance of phosphorylation events in key DNA topology–related proteins remains incompletely understood. This study aimed to investigate the prognostic significance of phosphorylation of TOP1, TOP2A, TOP2B, and C1orf35 in HCC and to characterize their associated molecular features to identify potential diagnostic and therapeutic biomarkers.

**Methods:**

Publicly available HCC phosphoproteomic and proteomic datasets were analyzed to identify significantly upregulated phosphorylation sites of TOP1, TOP2A, TOP2B, and C1orf35. Integrated bioinformatics and machine learning approaches were applied, including Gene Ontology (GO), Kyoto Encyclopedia of Genes and Genomes (KEGG), protein–protein interaction (PPI) network analysis, drug–gene interaction analysis, and survival analyses (overall and disease-free survival).

**Results:**

A total of 11,547 phosphorylation sites corresponding to 4043 phosphoproteins were quantified from 159 HCC patients. Phosphorylation of TOP1, TOP2A, TOP2B, and C1orf35 was significantly upregulated. Enriched pathways included DNA damage response, homologous recombination repair, cell cycle regulation, SUMOylation, and TP53 signaling. PPI analysis identified these proteins as highly interconnected hub nodes. Elevated expression was significantly associated with poor clinical outcomes.

**Conclusions:**

Phosphorylated TOP1, TOP2A, TOP2B, and C1orf35 are strongly associated with HCC progression and poor prognosis, highlighting their potential as prognostic biomarkers and therapeutic targets. These insights not only enhance our understanding of the complex molecular mechanisms underlying HCC but also offer promising avenues for the identification of novel therapeutic targets.

## 1 Introduction

Liver cancer remains a major global health challenge, standing as the fourth leading cause of cancer-related mortality. Hepatocellular carcinoma (HCC) represents around 85%–90% of primary liver cancers, with chronic hepatitis B virus (HBV) and hepatitis C virus (HCV) infections being the primary contributing factors [1]. Other contributing factors include alcohol misuse and metabolic syndrome. Although direct-acting antiviral therapy has successfully cured chronic HCV infections, current antiviral treatments for HBV primarily control the virus rather than eradicate it, impacting an estimated 292 million people globally [2]. Mass spectrometry (MS)-based proteomics is essential for evaluating global protein abundance and post-translational modifications, offering insights beyond those obtained through genomic analysis alone [3]. Combining sequencing with mass spectrometry (MS) provides a more comprehensive understanding, bridging the gap between cancer genotype and phenotype by leveraging functional proteomics and uncovering key signaling networks. In normal cells, around one-third of proteins are regulated through phosphorylation, a crucial post-translational modification that governs essential biological processes such as proliferation, cell division, apoptosis, and cell survival [3,4]. Proteins can transition between dephosphorylated and phosphorylated states, regulated by the coordinated actions of protein phosphatases and kinases. Protein phosphatases catalyze dephosphorylation, while protein kinases facilitate phosphorylation by targeting the hydroxyl groups of specific amino acid side chains [5]. HCC can often be diagnosed effectively and uniquely using multiphase cross-sectional imaging, reducing the need for invasive tissue sampling. Therefore, ongoing efforts aim to enhance screening, diagnosis, and treatment strategies to improve the prognosis of this aggressive malignancy [5]. Despite advancements, reliable biomarkers for early HCC detection and monitoring remain limited. Current biomarkers exhibit low sensitivity and inconsistent specificity, with varying cutoff points and limited effectiveness even in longitudinal assessments or biomarker combinations [6]. The exact causes and biomarkers of liver cancer remain unclear. The disease may be linked to genetic factors, chromosomal abnormalities, gene fusions, and other influences. As a result, conducting in-depth research into the molecular mechanisms of liver cancer is crucial [6].

Type 2 DNA topoisomerases (TOP2) are evolutionarily conserved enzymes and key biomarkers of cell proliferation. They play crucial roles in essential cellular processes, including DNA replication, transcription, and chromosome segregation. The human TOP2A isoform is highly expressed during mitosis and is vital for cell division. Its primary function is to regulate topological entanglements in DNA, preventing disruptions that could interfere with cell division or gene transcription [7]. The DNA topoisomerase 2-α (TOP2A) gene, located on human chromosome 17 (17q21-22), encodes the Top2αenzyme. This isoform is a critical target for various antineoplastic agents widely used in cancer chemotherapy. However, resistance can arise in cancer cells due to TOP2 point mutations, alterations in gene expression, or post-translational modifications (PTMs), limiting the effectiveness of these treatments [7].

Two distinct topoisomerases have been identified: Topoisomerase 1 (TOP1) and Topoisomerase 2 (TOP2), with TOP2A being the primary isoform of TOP2. Both TOP1 and TOP2A serve as key tumor drivers in various malignancies, making them highly promising targets for anticancer drug development [8]. Recent research has increasingly demonstrated that TOP2A is significantly overexpressed in tumor tissues (*p* < 0.01) and is negatively correlated with patient prognosis (*p* = 0.002). Numerous studies have identified TOP2A as both a prognostic biomarker and a potential therapeutic target in various cancers, including bladder urothelial carcinoma (BLCA), lung adenocarcinoma, prostate cancer, colon cancer, and breast cancer [9]. Additionally, TOP2A mRNA and protein have been found at elevated levels in hepatocellular carcinoma (HCC), suggesting that TOP2A may serve as a promising biomarker for HCC [10]. Several TOP2A inhibitors, including anthracyclines (doxorubicin, epirubicin) and epipodophyllotoxins (etoposide, teniposide), have been widely utilized in clinical settings [7]. However, traditional TOP2A inhibitors are often associated with severe side effects and suboptimal therapeutic outcomes. This underscores the urgent need to develop new, more effective TOP2A inhibitors with fewer side effects to improve cancer treatment outcomes [11]. The advancement of proteomic techniques has significantly accelerated the identification and characterization of post-translational modifications (PTMs) [12,13]. Due to their importance in cancer therapy, TOP2A PTMs have been predominantly studied in cancer cell lines, while their role in maintaining normal cell homeostasis has received comparatively less attention. Recently, researchers identified phosphorylation and acetylation sites in human TOP2A expressed in the yeast *S. cerevisiae* and a hamster mammalian cell line (BHK21), providing a foundational overview of modifications associated with the TOP2A isoform [14].

Topoisomerase IIβ (TOP2B) is a crucial regulator of chromatin topology. The trapping of TOP2B has long been recognized as a major contributor to DNA damage and genomic rearrangements, particularly in leukemia and prostate cancer [15]. Improperly repaired TOP2B-mediated DNA double-strand breaks can lead to chromosomal translocations, especially in genes essential for hematopoiesis, potentially driving leukemia development [16]. Recent studies have established a strong link between TOP2B activity and cancer progression, highlighting its role in oncogenesis [17]. Additionally, research has demonstrated that TOP2B is essential for B cell development. Mice deficient in TOP2B exhibit impaired B cell differentiation, with significantly reduced numbers of B cell precursors at various developmental stages in both the bone marrow and spleen [15]. This finding underscores the critical role of TOP2B in normal B cell maturation and suggests that its dysfunction may contribute to B cell-related disorders.

C1orf35, also known as MMTAG2, is a gene encoding a protein with currently undefined function. According to data from The Human Protein Atlas, C1orf35 exhibits low cell type specificity, indicating its expression across various cell types without significant enrichment in anyone [18]. Although C1orf35’s function remains largely unclear, recent studies have identified it as a potential oncogene that drives cell cycle progression from G1 to S phase [19]. Further investigation revealed that C1orf35 promotes cell proliferation by regulating c-MYC (v-myc myelocytomatosis viral oncogene homolog) expression, a key factor in tumor development. Interestingly, the oncogenic effects of C1orf35 can be reversed by inhibiting c-MYC, indicating that C1orf35 may contribute to disease progression via this pathway [18]. Moreover, C1orf35 has been shown to modulate c-MYC expression and restore its transcription in multiple myeloma (MM) patients and cell lines when suppressed by actinomycin D (Act D), underscoring its potential role in maintaining oncogenic signaling [19]. As of now, there is no information regarding the role of C1orf35 in hepatocellular carcinoma (HCC). Existing studies have not identified a direct association between C1orf35 and HCC development or progression. Further research is necessary to elucidate any potential involvement of C1orf35 in HCC and its underlying mechanisms.

Bioinformatics, a critical aspect of life sciences, has been leading advancements in life science and technology research. By uncovering the biological insights within big data, bioinformatics bridges the gap between data and clinical applications [20]. It plays a particularly essential role in cancer treatment, especially in the analysis and reporting of gene detection data. However, existing literature contains relatively few reports on the specific mechanisms of TOP1, TOP2A, and TOP2B in hepatocellular carcinoma (HCC), and no studies have been reported on C1orf35 in HCC [6]. Therefore, we aim to investigate the role and underlying mechanisms of TOP1, TOP2A, TOP2B, and Clorf35 in HCC through bioinformatics analysis. This research aims to provide a new scientific foundation and novel therapeutic insights for better understanding the pathogenesis of liver cancer and advancing biological treatments for tumors.

The relationship between TOP1, TOP2A, TOP2B, C1orf35, and liver cancer remains unclear, and no drugs currently available target the genes identified in this study. However, the findings suggest potential for drug development based on these results. This study aims to utilize bioinformatics to identify core genes associated with hepatocellular carcinoma (HCC) and normal tissues, followed by enrichment and pathway analysis. Public datasets will be employed to validate the significant role of TOP1, TOP2A, TOP2B, and C1orf35 in liver cancer, with further confirmation through basic cell experiments. The primary goal of this research is to explore the involvement of TOP1, TOP2A, TOP2B, and C1orf35 in liver cancer, applying these findings to clinical practice to better understand the disease and offer new avenues for the prevention and precision treatment of liver cancer.

Despite the abundance of available data, the mechanisms regulating the differential expression of TOP1, TOP2A, TOP2B, and C1orf35 in HCC remain largely unexplored. Moreover, the roles of TOP1, TOP2A, TOP2B, and C1orf35 phosphorylation in HCC have not yet been investigated, and to our knowledge, no prior studies have specifically focused on these genes. Therefore, the primary objective of this study is to evaluate whether TOP1, TOP2A, TOP2B, and C1orf35 gene expression could serve as novel biomarkers for HCC, offering new insights into its pathogenesis and potential therapeutic strategies. Moreover, it’s important to highlight that a comprehensive analysis of C1orf35 immunohistochemical expression in HCC has not been previously conducted. To date, the U.S. Food and Drug Administration (FDA) has approved over two dozen small-molecule protein kinase inhibitors and six therapeutic antibodies targeting protein kinases for clinical use, primarily focusing on targeted cancer therapies [21]. In addition to these FDA-approved treatments, numerous other protein kinase inhibitors are currently under investigation in clinical trials. Notably, a wide range of small-molecule compounds capable of modulating kinase activity, either by activation or inhibition have been developed, showing promising therapeutic potential for a variety of human diseases [22,23].

Alterations in the tightly regulated processes of protein phosphorylation and dephosphorylation, including their dysregulation or imbalance, play a critical role in the pathogenesis of numerous conditions, including cancer, neurodegenerative diseases, and metabolic disorders [1,24]. In this study, we aimed to investigate the roles of TOP1, TOP2A, TOP2B, and C1orf35 in HCC by integrating clinical data with advanced bioinformatics analyses. A retrospective analysis was conducted on 159 HCC patients to uncover insights into disease dynamics [25]. We compared gene expression levels between HCC and non-HCC tissues, leveraging bioinformatics tools and publicly available databases to assess clinical data and patient survival outcomes, stratified by high and low gene expression levels. To validate our findings, we further analyzed differential gene expression profiles between HCC and non-HCC tissues, strengthening the link between genetic variations and patient survival rates. Additionally, bioinformatics tools were utilized to identify potential pathways and molecular targets, providing insights into the roles of TOP1, TOP2A, TOP2B, and C1orf35 in HCC. The primary objective of this study was to systematically characterize the expression and phosphorylation profiles of TOP1, TOP2A, TOP2B, and C1orf35 in HCC and to evaluate their associations with clinical outcomes. We further aimed to elucidate the molecular pathways linked to these phosphorylation events and assess their potential as prognostic biomarkers and therapeutic targets.

## 2 Materials & Methods

### 2.1 Data Set Collection and Preprocessing

We obtained proteomic and phosphoproteomic datasets from the publicly available CPTAC database (https://dctd.cancer.gov), [26] which offers mass spectrometry-based discovery proteomics. Our study conducted a comparative analysis of clinical data and patient survival outcomes based on high and low protein expression levels (|log_2_ Fold Change (FC)| ≥ 1.0, adjusted *p* < 0.0001), utilizing bioinformatics tools and public databases. Additionally, we examined differential protein expression between HCC and non-HCC tissues. Patient survival rates (Kaplan-Meier) were further validated based on protein expression levels. To explore the potential pathways associated with these findings, we employed several bioinformatics tools, including R (version 4.3.3) with the limma package, DGIdb (version 4.2.0), STITCH (version 5.0), TargetScan (release 8.0), BioGPS, and cBioPortal. Notably, the CPTAC (https://proteomics.cancer.gov/cptac), TCPA (https://tcpaportal.org), and TCGA (https://www.cancer.gov/tcga) databases are publicly accessible and open source. This study followed the data access policies and publication guidelines of these databases, eliminating the need for approval from a local ethics committee.

### 2.2 Bioinformatics and Expression Analysis

The acquired datasets provide detailed information on phosphorylation site levels and total protein abundances across samples, obtained through TMT11plex labeling experiments [25]. After normalization, relative abundances were calculated as log2 ratios compared to pooled reference samples. Proteins were filtered based on a threshold of log_2_FC ≥ 1.0 and adjusted *p*-values < 0.0001, indicating significant upregulation. All data analyses were performed in RStudio using the Limma package. Limma applies linear models with empirical Bayes moderation to improve differential expression detection, particularly when sample sizes are small. Features with excessive missing values or low abundance were filtered prior to analysis. Differential expression was then assessed using Limma with empirical Bayes variance moderation. Limma also supports normalization and visualization tools, including volcano plot generation. Additionally, it was used to perform statistical testing of protein and phosphorylation site abundance after adjusting phosphorylation levels to their corresponding total protein levels [27].

### 2.3 Identification of Differentially Expressed Phosphorylation Sites

We conducted a paired *t*-test analysis using the LIMMA package within the R-Bioconductor environment to identify differentially expressed phosphorylation sites between tumor and corresponding non-tumor liver tissues [27]. To account for multiple hypothesis testing, we applied the Benjamini-Hochberg false discovery rate (FDR) correction to adjust *p*-values, reducing the risk of false positives. Significance thresholds were set at adjusted *p-values* < 0.0001 and log_2_FC ≥ 1.0 to identify statistically significant differences. This stringent filtering helped ensure the reliability of the results. Our analysis focused on TOP1, TOP2A, TOP2B, and C1orf35 proteins and their corresponding phosphorylation sites, all of which showed notable differences between tumor and non-tumor tissues, detailed in Table 1 and Supplementary Table S1 (Log Ratio Data). To visually present these findings, we generated volcano plots using the gplots package within RStudio.

**Table 1:** Significantly altered phosphoproteins and associated phosphorylation sites in hepatocellular carcinoma (HCC) (|log_2_Fold Change (FC)| ≥ 1.0; adjusted *p*-value < 0.0001), identified using the LIMMA package in RStudio

No.	Gene	Site	Peptide	Log_2_FC	Ave Expr	*p-*Value	adj. *p-*Value
1	*C1orf35*	S231	HHHHDsDsNSPCCK	2.9102	−0.0335	4.05 × 10^−23^	1.45 × 10^−22^
2	*C1orf35*	S233	HHHHDsDsNSPCCK	2.9100	−0.0335	4.05 × 10^−23^	1.45 × 10^−22^
3	*C1orf35*	S177	AEDQTEsSCESHR	1.5096	−0.1816	1.56 × 10^−31^	1.00 × 10^−30^
4	*C1orf35*	S217	RPAEATSsPTSPERPR	1.4009	−0.1219	3.62 × 10^−34^	2.87 × 10^−33^
5	*C1orf35*	T175	AEDQtEsSCESHR	1.2385	−0.0979	2.40 × 10^−26^	1.07 × 10^−25^
6	*TOP1*	S97	VRAsGDAK	2.2042	−0.4501	2.26 × 10^−33^	1.68 × 10^−32^
7	*TOP2A*	S1377	SVVsDLEADDVK	2.8393	−1.0437	2.17 × 10^−61^	1.93 × 10^−58^
8	*TOP2A*	S1106	VPDEEENEEsDNEKETEK	2.6018	−1.4385	6.09 × 10^−65^	1.17 × 10^−61^
10	*TOP2A*	S1351	TDDEDFVPsDASPPK	2.2055	−1.3968	2.70 × 10^−45^	7.05 × 10^−44^
11	*TOP2A*	S1354	TDDEDFVPSDAsPPK	2.0393	−1.1645	2.50 × 10^−28^	1.28 × 10^−27^
12	*TOP2A*	S1374	SVVsDLEADDVK	2.0386	−0.6726	2.93 × 10^−38^	3.39 × 10^−37^
13	*TOP2A*	S1213	TQMAEVLPsPR	1.5915	−0.3720	4.11 × 10^−40^	5.66 × 10^−39^
14	*TOP2A*	S1295	RNPWsDSESDR	1.1924	−0.8719	1.61 × 10^−31^	1.03 × 10^−30^
15	*TOP2B*	S1408	ASPITNDGEDEFVPs DGLDKDEYTFSPGK	1.2646	−0.5220	2.21 × 10^−28^	1.13 × 10^−27^
16	*TOP2B*	S1547	KASGsENEGDYNPGR	0.9550	0.0041	3.04 × 10^−40^	4.25 × 10^−39^

Note: TOP: Topoisomerase; Clorf35: Chromosome 1 Open Reading Frame 35.

### 2.4 Functional Annotation and Pathway Analysis of Differentially Regulated Phosphosites

A well-defined and methodical analytical workflow was implemented to facilitate robust interpretation and clear visualization of the data, allowing the functional significance of differentially regulated phosphorylation sites to be systematically explored. Gene Ontology (GO) was employed as a standardized resource to categorize gene product functions across species and to identify prominent biological features within large-scale omics datasets [28]. To further characterize these functional patterns, pathway enrichment analyses were combined with graphical representations [29], including heatmap visualizations, to emphasize the distribution of phosphosites across biological processes (BP), cellular components (CC), and molecular functions (MF). Using the Cluster Profiler R 4.3.3 package, we performed pathway enrichment analysis, combining it with Gene Set Enrichment Analysis (GSEA) to annotate and interpret the functional significance of these differentially expressed phosphorylation sites by linking them to established pathways and biological processes [30].

### 2.5 Overall Survival Analysis of the Hub Genes in HCC

Survival data from 159 HCC patients in the CPTAC dataset were analyzed using Kaplan-Meier survival curves and the log-rank test to evaluate correlations between candidate proteins and HCC outcomes (*p* ≤ 0.05). These analyses were carried out with the survival (version 3.5–7) and survminer (version 0.4.9) in R 4.3.3 packages. Patients were divided into high and low expression groups using a median cutoff of 50%. To visualize expression levels of the identified phosphorylation sites and their corresponding proteins, we employed the ggplot2 package. Additionally, proteomics data were integrated to further explore and validate phosphoproteomic findings. Proteins showing a significant (adj. *p value* ≤ 0.05 and log_2_FC ≥ 1.0) association with patient survival were selected for further analysis. All statistical analyses and data processing were conducted within RStudio.

### 2.6 Employing a Gene Signature as an Independent Prognostic Factor for Overall Survival

To evaluate whether the proposed prognostic model functions independently of other clinical and pathological characteristics, we performed both univariate and multivariate Cox regression analyses. Variables considered included age, sex, tissue source, pathological stage, T stage, and the HCC patient risk score, as shown in Table 2. In these analyses, the clinical features were treated as predictor variables, while overall survival (OS) was the outcome of interest. Hazard ratios (HRs) were computed along with 95% confidence intervals (CIs) and two-sided *p*-values to determine the statistical significance of each factor.

**Table 2:** Summary of hepatocellular carcinoma (HCC) patient data from the CPTAC database

Parameter	Mean	Coef	Exp (Coef)	Exp (-Coef)	Se (Coef)	Upper 0.95	Lower 0.95	Z Value	*p-*Value
**Sex**	N/A	0.330	1.39	0.72	0.34	2.68	0.72	0.983	0.326
**Age**	53.9	0.001	1.00	1.00	0.01	1.02	0.98	0.068	0.840
**AFP**	6795.5	0.000	1.00	1.00	0.00	1.00	1.00	0.403	0.687
**ALB**	40.49	0.032	1.03	0.97	0.04	1.11	0.96	0.836	0.403
**PTT**	12.01	−0.003	0.99	1.00	0.07	1.13	0.88	−0.040	0.967
**ALT**	50.22	0.000	1.00	1.00	0.003	1.00	0.995	−0.105	0.917
**TB**	12.02	−0.047	0.95	1.05	0.03	1.02	0.899	−1.566	0.117
**GGT**	79.71	−0.001	1.00	1.00	0.002	1.00	0.996	−0.365	0.715
**Tumor Size (cm)**	6.57	0.056	1.01	0.945	0.04	1.15	0.98	1.398	0.162
**Tumor differentiation**	2.39	−0.166	0.847	1.18	0.258	1.40	0.51	−0.644	0.519
**Medical history of liver cirrhosis**	N/A	−0.563	0.569	1.76	0.280	0.984	0.329	−2.015	0.044
**No of tumors**	1.64	−0.044	0.96	1.045	0.111	1.191	0.77	−0.396	0.692

Note: N/A: Not applicable, GGT: Gamma-glutamyl transferase; TB: Total bilirubin; ALT: Alanine aminotransferase; PTT: Partial thromboplastin time; ALB: Albumin; AFP: Alpha-fetoprotein.

### 2.7 Validation of the Gene Signature across Multiple Databases

We conducted mRNA expression profiling of the gene signature in hepatocellular carcinoma (HCC) and normal liver tissues using the Oncomine database (http://www.oncomine.org). Analyses were performed under specific criteria: *p*-value < 0.001, fold change > 2, and gene ranking within the top 10%. Datasets were carefully chosen based on statistical significance, taking into account sample size, fold change, *t*-test results, type of analysis, and *p*-values. To assess protein-level expression associated with the gene signature, immunohistochemical images were obtained from the Human Protein Atlas (http://www.proteinatlas.org), further strengthening validation [31]. For additional verification, an independent HCC cohort was sourced from the International Cancer Genome Consortium (ICGC) [32]. Gene signature expression levels were extracted and compared between tumor and non-tumor tissues. Statistical differences were evaluated using the Wilcoxon signed-rank test, with two-sided *p*-values < 0.001 considered significant. Moreover, data from the Human Protein Atlas provided complementary evidence of differential expression for key genes between HCC and normal tissues, reinforcing the reliability of our results.

### 2.8 Creation and Assessment of Nomograms for Predicting (HCC) Survival

Nomograms are powerful tools for predicting cancer patient outcomes, transforming complex statistical models into straightforward, user-friendly visual charts that help estimate an individual’s overall survival (OS) [33]. In this study, we combined all independent clinical and pathological prognostic factors identified through Cox regression analysis to build a comprehensive nomogram. This model predicts OS probabilities at 1, 3, and **5** years for HCC patients, as shown in Supplementary Table S2 (OS probabilities over 5 years). To evaluate the nomogram’s reliability, we compared the predicted survival probabilities with the actual outcomes using a calibration curve, where closer alignment with the reference line signifies higher accuracy. We conducted time-dependent ROC analysis to account for censored survival data, using the timeROC package in R 4.3.3. Additionally, Receiver Operating Characteristic (ROC) analysis was conducted to compare the predictive performance of the integrated nomogram against individual nomograms based on each clinical and pathological factor, determining whether the combined model provided a more accurate survival prediction.

### 2.9 Hub Genes and Drug Interaction Analysis

We identified drugs targeting hub genes associated with hepatocellular carcinoma (HCC) using the Drug Gene Interaction Database (DGIdb v3.0.2) (https://dgidb.org) on 02 September 2025, [34] a platform that compiles drug-gene interaction data from over 30 reliable sources, including Ensembl, ChEMBL, DrugBank, PubChem, NCBI Entrez, clinical trial databases, PharmGKB, and NCBI PubMed literature references. To ensure the credibility of our results, we prioritized drugs supported by multiple databases or validated through PUBMED references. Additionally, only FDA-approved drugs were selected for further investigation. For a more comprehensive understanding of drug interactions, we used the Search Tool for Interacting Chemicals (STITCH v5.0) to visualize the hub gene interaction network with high confidence (0.700), limiting the network to no more than 10 interactions. The visualization was generated using the default settings. STITCH integrates data from over 430,000 chemicals, enabling the mapping of potential chemical-gene associations and the identification of additional drug interactions relevant to HCC treatment [35].

### 2.10 Comparative Toxicogenomic Database (CTD) Analysis

The Comparative Toxicogenomics Database (CTD) (https://ctdbase.org/) compiles extensive data on chemical substances, genes, functional phenotypes, and disease interactions, offering valuable insights into disease-related environmental exposures and potential drug mechanisms [36]. We conducted searches using the official gene symbols of our target genes, applied filters to include only human data, and considered associations supported by at least two independent studies. Redundant or overlapping disease associations were consolidated to avoid duplication, and only statistically significant or high-confidence relationships were retained for downstream analysis. These steps ensured that the reported disease associations were accurate, relevant, and non-redundant. In this study, we input the core genes into the CTD platform to identify the most strongly associated diseases and used Excel to generate a radar chart visualizing the differential expression of each gene, as shown in Supplementary Table S3 (CTD analysis).

### 2.11 The miRNA

TargetScan (www.targetscan.org) is an online database designed for predicting and analyzing miRNA-target gene interactions [37]. In this study, we utilized TargetScan release 8.0, queried in September 2021, to identify miRNAs that regulate differentially expressed genes (DEGs), thereby providing insights into potential post-transcriptional regulatory mechanisms.

### 2.12 BioGPS Database Analysis

We utilized the BioGPS database (http://biogps.org/) to analyze TOP1, TOP2A, TOP2B, and C1orf35 gene expressions across normal human tissues. By entering TOP1, TOP2A, TOP2B, and C1orf35 in the search box and selecting “Human” as the species, we retrieved gene expression profiles to assess their distribution in various tissues [38]. In our study, BioGPS expression data for the genes were retrieved and processed as follows: raw expression values were log2-transformed, normalized across samples using quantile normalization, and finally summarized by calculating the mean expression for each tissue type.

### 2.13 c-BioPortal Database Analysis

c-BioPortal V6.4.1 (http://cbioportal.org) is an open-access platform for exploring, visualizing, and analyzing multi-dimensional cancer genomic data, encompassing 225 cancer studies [39]. In this study, we utilized c-BioPortal to investigate TOP1, TOP2A, TOP2B, and C1orf35 gene mutations in TCGA LIHC (Liver Hepatocellular Carcinoma) samples, analyzing mutation frequency, types, and their potential impact on tumor progression. Mutation frequencies and types were obtained from TCGA datasets within cBioPortal and categorized as missense, nonsense, frameshift, splice site, or other variant types. To evaluate the potential functional impact of these mutations, predictive tools such as SIFT and PolyPhen-2 were employed to assess the likelihood of deleterious effects on protein function. In addition, mutation recurrence was examined, and findings were cross validated across independent datasets to enhance the reliability of the results.

### 2.14 Univariate Cox Regression Analysis

Using the Survival package in R, we conducted a univariate Cox regression analysis to identify differentially methylated genes (DMGs) associated with patient outcomes. This analysis computed hazard ratios (HRs) along with their 95% confidence intervals (CIs) to assess the impact of each gene on survival. A *p*-value threshold of < 0.05 was used to determine statistical significance. In addition to univariate Cox regression, we performed multivariate Cox regression analysis, including key clinical variables such as age, gender, tissue source, pathological stage, and T stage. This approach allowed us to account for potential clinical confounders and to evaluate whether the differentially methylated genes (DMGs) act as independent prognostic factors.

### 2.15 TIMER3 Database Analysis

The Tumor Immune Estimation Resource (TIMER3) database (https://compbio.cn/timer3//) contains data from 32 cancer types and 10,897 tissue samples derived from the TCGA database [40]. It enables the systematic analysis of correlations between tumors, immune cell infiltration, and tumor-related gene expression [41]. Additionally, TIMER provides insights into the relationships between gene expression, prognosis, gene mutations, and copy number variations in cancer patients [41]. In this study, we utilized TIMER3 to investigate the expression and prognostic significance of TOP1, TOP2A, TOP2B, and C1orf35 across various cancer types. Specifically, we examined the correlation between TOP1, TOP2A, TOP2B, and C1orf35 expression and immune cell infiltration, including B cells, CD4^+^ T cells, CD8^+^ T cells, Neutrophils, Macrophages, and Eosinophil cells, in Liver hepatocellular (LIHC) patients. Furthermore, using the Gene module in TIMER3, we analyzed the association between TOP1, TOP2A, TOP2B, and C1orf35 expressions and the surface markers of immune cells, providing insights into its potential role in tumor immune microenvironment regulation.

TIMER3 generates heatmaps showing the log-fold changes in immune infiltration levels between tumors with the input gene mutated and tumors without mutations. Significance is indicated as follows: higher levels in mutants (*p* < 0.05, log_2_FC > 0), lower levels in mutants (*p* < 0.05, log_2_FC < 0), higher levels in alterations (*p* < 0.05, log_2_FC > 0), lower levels in alterations (*p* < 0.05, log_2_FC < 0), increased risk (*p* < 0.05, z > 0), and decreased risk (*p* < 0.05, z < 0). Moreover, TIMER3 generates a heatmap showing the purity-adjusted Spearman’s correlation (rho) across multiple cancer types. By clicking on a specific cell in the heatmap, a scatter plot is displayed to illustrate the relationship between immune infiltration estimates and gene expression. We recommend selecting the “Purity Adjustment” option, which uses partial Spearman’s correlation to account for tumor purity. Positive correlations are indicated by (*p* < 0.05, ρ > 0) and negative correlations by (p < 0.05, ρ < 0).

### 2.16 Extracting Data from Kaplan Meier-Plotter Database

The Kaplan-Meier Plotter database (http://kmplot.com/analysis/) is a powerful tool for analyzing the clinical significance and prognostic value of gene expression across various cancers [42]. It evaluates patient survival outcomes based on mRNA expression levels. In this study, we utilized the Kaplan-Meier Plotter to assess the prognostic impact of TOP1, TOP2A, TOP2B, and C1orf35 in liver cancer by analyzing. Overall Survival (OS), Recurrence-Free Survival (RFS), Progression-Free Survival (PFS), and Disease-Specific Survival (DSS). These analyses provide valuable insights into the correlation between TOP1, TOP2A, TOP2B, and C1orf3**5** expression levels and liver cancer prognosis, helping to establish their potential as prognostic biomarkers.

### 2.17 Protein–Protein Interaction Network and Identification of Hub Genes

To explore the interactions among differentially expressed proteins at the protein level, we employed the STRING database to build a protein–protein interaction (PPI) network. Understanding these interactions is crucial for elucidating the molecular and metabolic mechanisms underlying tumor development. The network encompassed 4 significant proteins along with their phosphorylation sites, using a confidence score threshold of 200 at the protein level. Differentially expressed genes (DEGs) were mapped to the PPI network via STRING, and the resulting network was visualized using R software (version 4.2.2).

### 2.18 Data Analysis

Clinical characteristics and patient survival outcomes were analyzed by stratifying samples into high- and low-protein expression groups (|log_2_ fold change| ≥ 1.0, adjusted *p*-value < 0.0001). Differential protein expression analyses were performed to compare hepatocellular carcinoma (HCC) tissues with non-tumor liver samples. Survival outcomes were evaluated using Kaplan–Meier analysis based on protein expression levels. All statistical and bioinformatics analyses were conducted using R software (version 4.3.3) with the Limma package. Functional and interaction analyses were performed using DGIdb (version 4.2.0), STITCH (version 5.0), TargetScan (release 8.0), BioGPS, TIMER3, CTD, STRING, and cBioPortal. Proteomic, transcriptomic, and clinical data were obtained from publicly available databases, including CPTAC, TCPA, and TCGA.

## 3 Results

### 3.1 CPTAC Patient Characteristics

We retrieved data from the CPTAC database, encompassing phosphoproteomics and proteomics datasets along with clinical information from 159 patients treated in the Department of Hepatobiliary Surgery [25]. This dataset served as the foundation for our proteogenomic analysis. The collected clinical parameters included gender, age, family history of liver cancer, liver cirrhosis status, Alpha-fetoprotein (AFP) levels, tumor size, tumor count, and various biochemical markers (such as total bilirubin, PTT, GGT, TB, ALT, and albumin), as detailed in Supplementary Table S4 (Patient Demographics and Clinical Data). All patients were systematically monitored throughout the study until either death or loss to follow-up, in accordance with the study protocol. This comprehensive dataset enabled an in-depth exploration of the proteogenomic landscape, with a particular emphasis on TOP1, TOP2A, TOP2B, and C1orf35 proteins and their associations with clinical parameters.

### 3.2 The Clinical Application in HCC

Table 2 provides a comparative assessment of the general clinical characteristics between the two participant groups. The analysis encompassed variables such as gender, liver cirrhosis status, age, Alpha-fetoprotein (AFP) levels, total bilirubin (TBil) levels, tumor size, tumor count, TB, PTT, GGT, alanine aminotransferase (ALT) levels, and albumin (ALB). No statistically significant differences were observed across these parameters (all *p* > 0.05), indicating similar distributions between the groups. However, a significant difference was identified in the family history of liver cancer (*p* < 0.05), suggesting a potential role of familial predisposition. Additionally, the correlation between age and gender among HCC patients, yielding a *p*-value of 0.84. This non-significant result implies that gender alone does not have a substantial impact on prognosis within this dataset. While a slight trend toward improved survival probability in females was noted, it did not reach statistical significance

### 3.3 Detection of Aberrantly Regulated Phosphorylation Sites through Deep Phosphoproteomic Profiling

**Figure 1:**
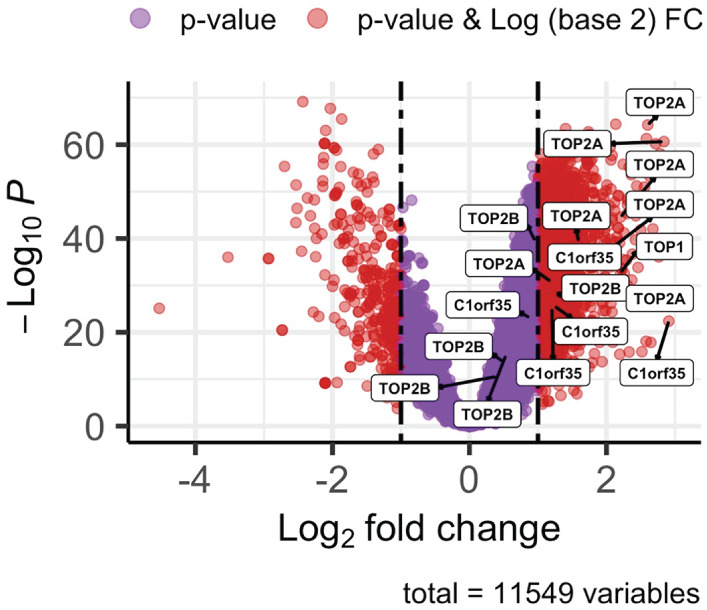
Volcano plot illustrating differential gene expression in hepatocellular carcinoma (HCC) samples. Four genes with corresponding phosphosites (|log_2_ Fold Change (FC)| ≥ 1.0, *p-*value < 0.001) are labeled by gene name. Downregulated genes are shown in red on the left, while upregulated genes are depicted in red on the right

An extensive phosphoproteomic evaluation was performed on 159 matched pairs of tumor and adjacent non-malignant tissues, resulting in the identification of 11,549 phosphorylation sites distributed across 4043 phosphoproteins. Quantitative measurements were available for 9994 phosphosites in at least 50% of the analyzed samples. Among these, 8198 sites showed elevated phosphorylation levels in tumor tissues, whereas 1796 sites displayed reduced abundance relative to their corresponding non-tumor samples. Statistical comparisons were carried out using a paired, two-tailed Student’s *t*-test, with multiple-testing correction applied using the Benjamini–Hochberg procedure (adjusted *p* < 0.0001). Further filtering identified 18 phosphosites mapping to four distinct genes that exhibited highly significant differences (*p* < 0.001) between tumor and non-tumor groups. Data visualization was performed in RStudio, where volcano plots and heatmaps were generated to clearly represent the observed phosphorylation patterns. Notably, four genes were markedly upregulated, as illustrated in the volcano plot shown in Fig. 1. To ensure statistical rigor in identifying differentially expressed proteins within the CPTAC database, we applied stringent criteria of log_2_FC ≥1.0 and *adj p-value* < 0.0001.

### 3.4 Functional Annotation, Pathway Exploration, and Visual Representation

**Figure 2:**
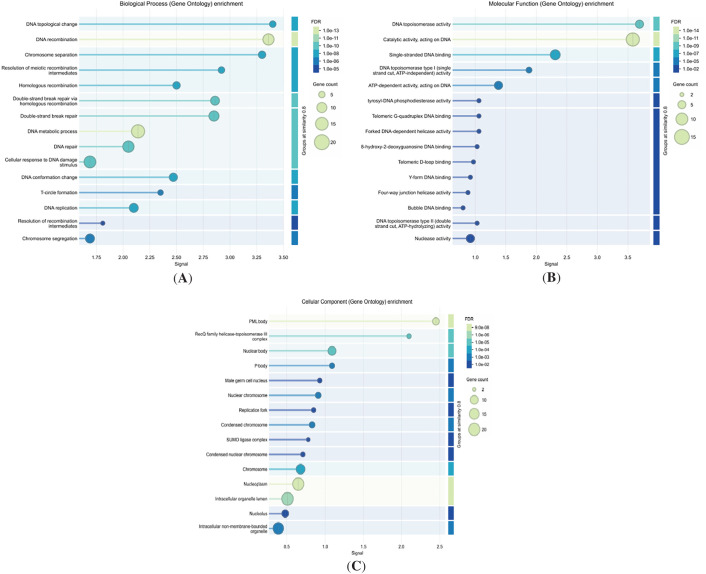
Gene ontology (GO) enrichment analysis of significant genes associated with the high-risk group. The analysis highlights key enriched (**A**) Biological processes (BP) and (**B**) Molecular functions (MF), and (**C**) Cellular component (CC), providing insights into the functional roles of these genes in HCC progression

We conducted a comprehensive KEGG pathway analysis of the identified phosphoproteins, encompassing 8198 upregulated and 1796 downregulated phosphorylation sites, using Over-Representation Analysis (ORA) via the ClusterProfiler package in RStudio. Additionally, Gene Set Enrichment Analysis (GSEA) was performed for both Gene Ontology (GO) and KEGG pathways, with a specific focus on the upregulated genes TOP1, TOP2A, TOP2B, and C1orf35, along with their associated differentially expressed genes (DEGs). The GO enrichment analysis revealed significant involvement in key biological processes (BP), including DNA repair, T-circle formation, DNA metabolic process, cellular response to DNA damage stimulus, and chromosome segregation. Molecular function (MF) analysis identified enrichment in activities such as catalytic activity acting on DNA, single-stranded DNA binding, ATP-dependent activity, DNA topoisomerase activity, nuclease activity, and forked DNA-dependent helicase activity. These findings are presented in Fig. 2. Furthermore, cellular component (CC) analysis demonstrated that the phosphoproteins were predominantly localized within the chromosome, nucleoplasm, nucleolus, intracellular organelle lumen, nuclear body, and intracellular non-membrane-bounded organelle, as illustrated in Fig. 2.

**Figure 3:**
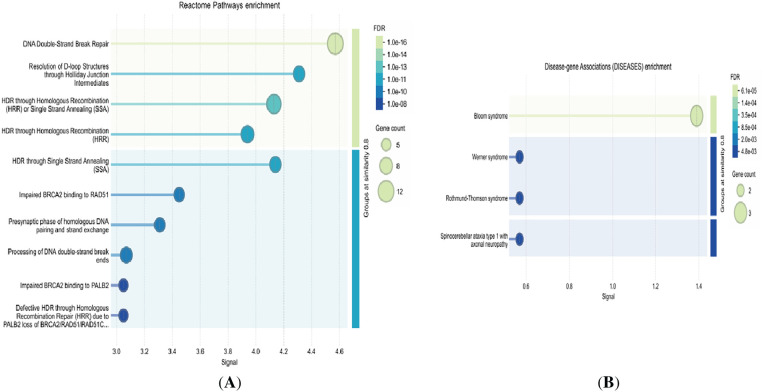
(**A**) Reactome pathway enrichment analysis of significantly upregulated phosphoproteins, highlighting the most relevant signaling pathways implicated in hepatocellular carcinoma. (**B**) Disease-gene association analysis for genes in the high-risk group within the red module, revealing key pathological links and potential disease mechanisms

KEGG pathway analysis revealed significant enrichment in pathways such as Homologous Recombination and the Fanconi Anemia Pathway, both of which are pivotal in maintaining genomic stability. Complementary analysis using WikiPathways further identified key DNA damage response mechanisms, including the DNA IR-damage and cellular response via ATR and DNA IR-double strand breaks and cellular response via ATM. Functional enrichment through Gene Set Enrichment Analysis (GSEA) of Reactome pathways highlighted several critical biological processes, such as DNA Double-Strand Break Repair, Homology-Directed Repair (HDR) through Homologous Recombination (HRR), SUMOylation, G2/M DNA damage checkpoint, Cell Cycle regulation, and Post-translational protein modification, including the Regulation of TP53 Activity through Phosphorylation and Impaired BRCA2 binding to RAD51 (Fig. 3A and Table 3). To further explore disease-gene associations for the four upregulated genes (TOP1, TOP2A, TOP2B, and C1orf35), we identified strong links to genetic disorders such as Bloom syndrome, Werner syndrome, Rothmund-Thomson syndrome, Spinocerebellar ataxia type 1 with axonal neuropathy, and liver cirrhosis, as illustrated in Fig. 3B. Additionally, we employed the pmcplot function to visualize trends in publication frequency across PubMed Central, enabling us to prioritize biologically and clinically relevant pathways. This bibliometric analysis is depicted in Fig. 4 and illustrates the number and proportion of publications associated with each pathway. Together, these multi-layered analyses provide robust insight into the functional significance of phosphorylated proteins in hepatocellular carcinoma (HCC) progression and their potential association with both inherited syndromes and liver disease.

**Table 3:** Reactome pathways identified via STRING network analysis of significant genes, conducted using RStudio

# Term ID	Term description	Observed gene count	Background gene count	Signal	False discovery rate	Matching proteins in network (Labels)
HSA-5693532	DNA Double-Strand Break Repair	12	149	4.57	3.28 × 10^−16^	TOPBP1, SLX4, WRN, MUS81, RMI1, TOP2A, TDP1, BLM, UBE2I, RAD52, EXO1, TDP2
HSA-5693567	HDR through Homologous Recombination (HRR) or Single Strand Annealing (SSA)	10	113	4.13	1.27 × 10^−13^	TOPBP1, SLX4, WRN, MUS81, RMI1, TOP2A, BLM, UBE2I, RAD52, EXO1
HSA-5685942	HDR through Homologous Recombination (HRR)	8	66	3.94	8.51 × 10^−12^	TOPBP1, SLX4, WRN, MUS81, RMI1, TOP2A, BLM, EXO1
HSA-5693568	Resolution of D-loop Structures through Holliday Junction Intermediates	7	32	4.31	8.59 × 10^−12^	SLX4, WRN, MUS81, RMI1, TOP2A, BLM, EXO1
HSA-5685938	HDR through Single Strand Annealing (SSA)	7	37	4.14	1.60 × 10^−11^	TOPBP1, WRN, RMI1, TOP2A, BLM, RAD52, EXO1
HSA-2990846	SUMOylation	9	172	2.95	1.11 × 10^−10^	TOP2B, WRN, BLM, UBE2I, RAD52, TOP1, RWDD3, SMC6, TOP2A
HSA-9709570	Impaired BRCA2 binding to RAD51	6	34	3.45	1.41 × 10^−9^	TOPBP1, WRN, RMI1, TOP2A, BLM, EXO1
HSA-5693607	Processing of DNA double-strand break ends	7	80	3.07	1.72 × 10^−9^	TOPBP1, WRN, RMI1, TOP2A, BLM, UBE2I, EXO1
HSA-5693616	Presynaptic phase of homologous DNA pairing and strand exchange	6	39	3.31	2.48 × 10^−9^	TOPBP1, WRN, RMI1, TOP2A, BLM, EXO1
HSA-3108232	SUMO E3 ligases SUMOylate target proteins	8	166	2.58	2.98 × 10^−9^	TOP2B, WRN, BLM, UBE2I, RAD52, TOP1, SMC6, TOP2A
HSA-9701192	Defective homologous recombination repair (HRR) due to BRCA1 loss of function	5	24	3.05	2.53 × 10^−8^	WRN, RMI1, TOP2A, BLM, EXO1
HSA-9704331	Defective HDR through Homologous Recombination Repair (HRR) due to PALB2 loss of BRCA1 binding function	5	24	3.05	2.53 × 10^−8^	WRN, RMI1, TOP2A, BLM, EXO1
HSA-9704646	Defective HDR through Homologous Recombination Repair (HRR) due to PALB2 loss of BRCA2/RAD51/RAD51C binding function	5	24	3.05	2.53 × 10^−8^	WRN, RMI1, TOP2A, BLM, EXO1
HSA-9709603	Impaired BRCA2 binding to PALB2	5	24	3.05	2.53 × 10^−8^	WRN, RMI1, TOP2A, BLM, EXO1
HSA-5693554	Resolution of D-loop Structures through Synthesis-Dependent Strand Annealing (SDSA)	5	26	3.01	2.82 × 10^−8^	WRN, RMI1, TOP2A, BLM, EXO1
HSA-69473	G2/M DNA damage checkpoint	6	77	2.58	5.57 × 10^−8^	TOPBP1, WRN, RMI1, TOP2A, BLM, EXO1
HSA-6804756	Regulation of TP53 Activity through Phosphorylation	6	92	2.37	1.47 × 10^−7^	TOPBP1, WRN, RMI1, TOP2A, BLM, EXO1
HSA-3108214	SUMOylation of DNA damage response and repair proteins	5	75	2	3.16 × 10^−6^	WRN, BLM, UBE2I, RAD52, SMC6
HSA-4615885	SUMOylation of DNA replication proteins	4	45	1.77	2.71 × 10^−5^	TOP2B, UBE2I, TOP1, TOP2A
HSA-1640170	Cell Cycle	8	658	0.99	5.62 × 10^−5^	TOPBP1, WRN, RMI1, TOP2A, BLM, UBE2I, EXO1, TOP2A
HSA-597592	Post-translational protein modification	10	1405	0.7	0.00019	BTBD1, TOP2B, WRN, BLM, UBE2I, RAD52, TOP1, RWDD3, SMC6, TOP2A
HSA-3065678	SUMO is transferred from E1 to E2 (UBE2I, UBC9)	2	6	1.11	0.0027	UBE2I, RWDD3
HSA-1500620	Meiosis	3	88	0.75	0.0111	TOP2A, BLM, UBE2I
HSA-174414	Processive synthesis on the C-strand of the telomere	2	19	0.74	0.0177	WRN, BLM
HSA-212436	Generic Transcription Pathway	7	1215	0.41	0.0267	TOPBP1, WRN, RMI1, TOP2A, BLM, UBE2I, EXO1

**Figure 4:**
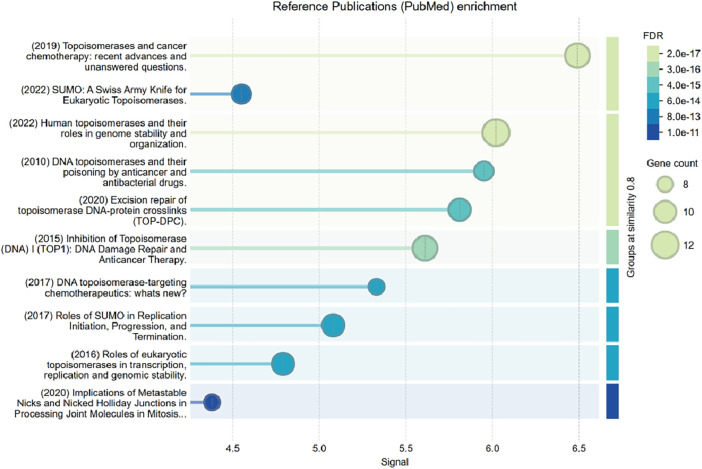
PubMed central (PMC) plot displaying the enrichment analysis results for significant proteins. The bubble plot illustrates significantly enriched literature themes related to topoisomerase activity, DNA repair, and cancer biology. Bubble size represents gene count, color indicates false discovery rate (FDR), and the *x*-axis shows the enrichment signal score

### 3.5 Protein-Protein Interaction (PPI) Network and Identification of Genes Hub

**Figure 5:**
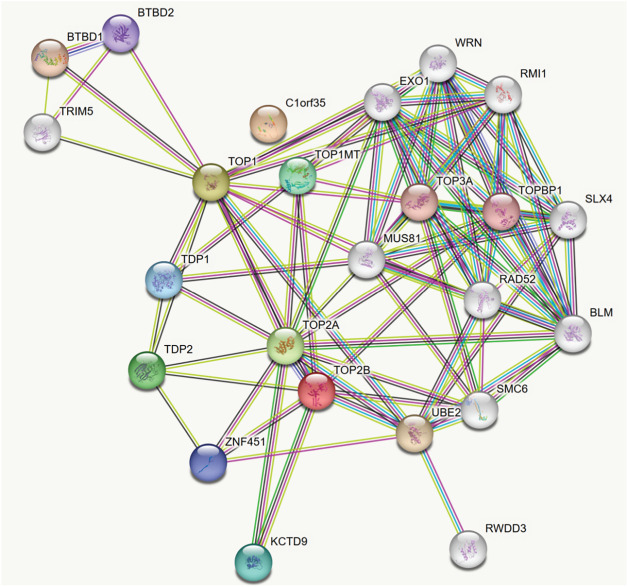
Protein–protein interaction (PPI) network of upregulated significant phosphoproteins. Connection line thickness represents betweenness centrality, while node color gradients correspond to log_2_FC. Green nodes indicate co-expression relationships based on data from the STRING database

To investigate the functional interactions among differentially expressed proteins, we utilized the Search Tool for the Retrieval of Interacting Genes/Proteins (STRING) (https://string-db.org/) to construct a protein-protein interaction (PPI) network [43]. Mapping these interactions is critical for unraveling the metabolic and molecular mechanisms driving tumor progression. Our analysis centered on four key proteins and their respective phosphosites, using a STRING confidence score threshold of 200 at the protein level. As shown in Fig. 5, the resulting network consisted of 24 nodes and 90 edges, significantly exceeding the 22 expected interactions (PPI enrichment *p*-value < 1.0 × 10^−16^), indicating a non-random pattern of protein associations. The network was visualized using RStudio, enabling a clearer depiction of the protein interconnectivity. Notably, TOP1, TOP2A, TOP2B, and C1orf35 emerged as central hub genes, each exhibiting high connectivity scores and pivotal roles in the network. Detailed information on these hub genes, including their interaction scores, false discovery rate (FDR), *p*-values, and associated enriched pathways identified through STRING analysis, is provided in Table 3.

### 3.6 Kinase Substrate Enrichment Analysis (KSEA)

**Figure 6:**
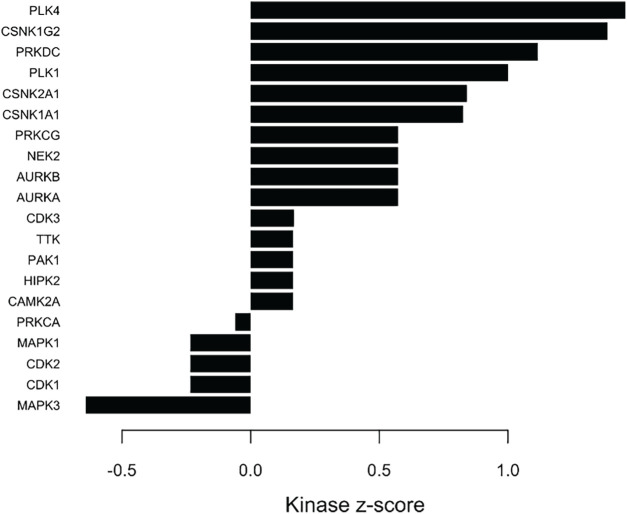
Kinase–substrate enrichment analysis (KSEA) highlighting differential kinase activity

We applied Kinase Substrate Enrichment Analysis (KSEA), a method that predicts changes in kinase activity by integrating the differential phosphorylation behavior of each kinase’s known substrate sites. As illustrated in Fig. 6, the enrichment results focus exclusively on kinase-specific phosphosites that met the statistical significance threshold in our dataset. By aggregating the direction and magnitude of fold-change values across all validated substrates, KSEA generates a consolidated enrichment score and significance estimate, enabling the identification of kinases whose targets exhibit coordinated and meaningful regulation under the experimental conditions.

The KSEA bar plot illustrates the predicted activity changes of multiple kinases based on the phosphorylation levels of their known substrates. Each bar represents a kinase score, reflecting the extent to which its substrates are significantly up or downregulated within the dataset. Positive bars indicate increased kinase activity, whereas negative bars suggest reduced activity. The magnitude of each bar corresponds to the strength of enrichment, enabling rapid identification of kinases that may be functionally activated or suppressed. In this figure, kinases with the highest positive scores are likely key regulators driving the observed phosphorylation changes, while those with strongly negative scores may represent inhibited signaling pathways. Overall, the plot provides a concise visual summary of the major signaling kinases implicated in the dataset. KSEA was performed to determine kinase substrate relationships, with the resulting enriched associations presented in Supplementary Table S5 (KSEA).

All identified serine, threonine, and tyrosine phosphorylation sites were scored and analyzed using KSEA. In addition to examining individual phosphosite changes, KSEA utilizes the mean log_2_ fold change of known substrates for a given kinase to systematically infer kinase activity. Based on the phosphoproteomic dataset, KSEA predicted altered activity for several kinases, including PLK4, AURKA, AURKB, CAMK2A, CSNK1A1, CSNK1G2, CSNK2A1, HIPK2, NEK2, PAK1, PAK4, PRKCG, PRKDC, TTK, and CDK3 (all with positive z-scores), as well as MAPK1, MAPK3, CDK1, CDK2, and PRKCA (all with negative z-scores), as illustrated in Fig. 6.

The kinase–substrate table provides detailed evidence of the specific phosphosites contributing to the activity changes observed in the KSEA bar plot. Several substrates exhibited strong and significant phosphorylation alterations, particularly those associated with C1orf35, TOP1, TOP2A, and TOP2B indicating substantial modulation of signaling pathways linked to chromatin dynamics, DNA topology, and transcriptional stress responses. Phosphosites on TOP2A, such as S1377, S1106, and S1351, showed high positive log_2_ fold changes and extremely low *p*-values, contributing prominently to the positive enrichment scores for kinases involved in regulating DNA replication and repair machinery. Likewise, multiple phosphorylation events on C1orf35 (S177, S217, S231, and S233) demonstrated coordinated regulation, suggesting that this protein may act as a key downstream node for several kinases highlighted in the KSEA analysis.

### 3.7 Time-Dependent ROC Curves and Kaplan-Meier Analysis Based on a Gene Signature

**Figure 7:**
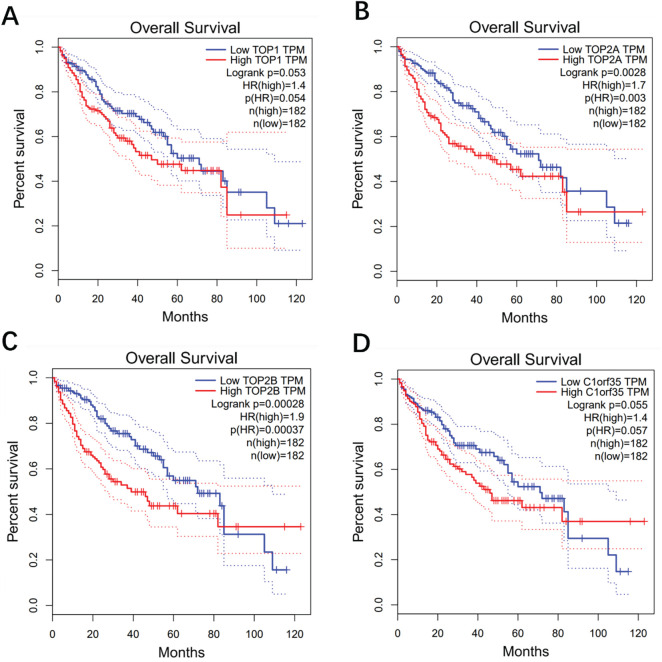
Kaplan-Meier overall survival plot analysis shows that the group with higher levels of 4 upregulated proteins; (**A**) TOP1, (**B**) TOP2A, (**C**) TOP2B, and (**D**) C1orf35 had a worse prognosis compared to the group with lower levels (*p* < 0.05). The dashed lines indicate the upper and lower confidence intervals

We employed Kaplan-Meier survival curve analysis to assess overall survival (OS) across two groups stratified by their median risk scores. To further evaluate the prognostic significance of the gene signature, we calculated the area under the time-dependent receiver operating characteristic (ROC) curve (AUC), where higher AUC values indicate superior predictive accuracy. Our findings revealed a statistically significant difference in OS between the high-risk and low-risk groups (*p* ≤ 0.05). Notably, elevated expression levels of TOP1, TOP2A, TOP2B, and C1orf35 were associated with poorer overall survival compared to lower expression levels (*p* ≤ 0.05) as depicted in Fig. 7. Further survival analysis demonstrated that High expression of TOP2A is significantly associated with poorer overall survival in this patient cohort. Patients with high TOP2A expression have a 1.7-fold increased risk of death compared to those with low expression (HR = 1.7, *p* = 0.003). These results suggest that TOP2A may serve as a negative prognostic biomarker, indicating worse outcomes when highly expressed.

**Figure 8:**
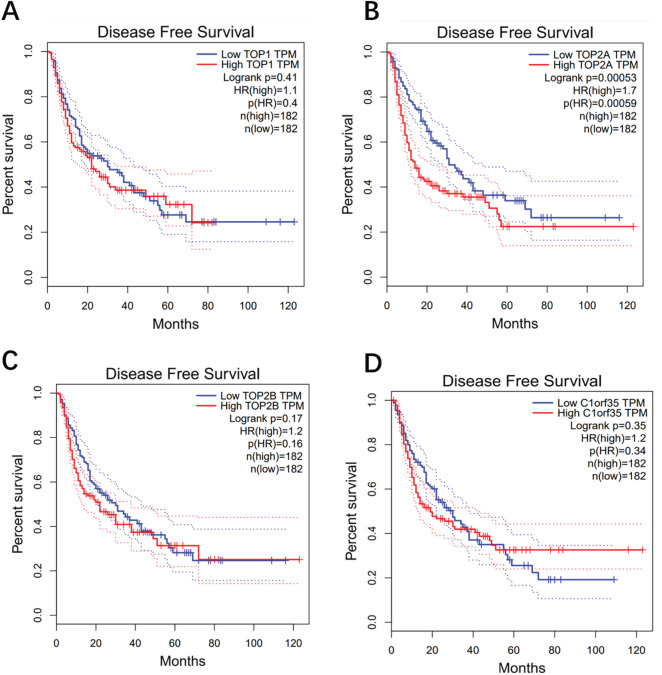
Kaplan-Meier disease-free survival plot analysis shows that the group with higher levels of 4 proteins: (**A**) TOP1, (**B**) TOP2A, (**C**) TOP2B, and (**D**) C1orf35 had a worse prognosis compared to the group with lower levels (*p* < 0.05). The dashed lines represent the upper and lower confidence intervals

Lower expression levels of TOP1 (HR = 1.4; *p* = 0.053), TOP2A (HR = 1.7; *p* = 0.003), TOP2B (HR = 1.9; *p* = 0.00037), and C1orf35 (HR = 1.40; *p* = 0.057) were significantly associated with improved overall survival (OS) in hepatocellular carcinoma (HCC) patients. These results suggest that high expression of these genes at diagnosis may serve as unfavorable prognostic indicators, contributing to poorer (OS) outcomes. For disease-free survival (DFS) analysis, patients were divided into low and high-expression groups based on a 50% expression cutoff. As shown in Fig. 8, lower gene expression levels were significantly linked to better DFS outcomes (*p* < 0.05), particularly for TOP2A (HR = 1.7; *p* = 0.00059), which demonstrated a strong association with improved DFS. In contrast, lower expression levels of TOP1 (HR = 1.1; *p* = 0.4), TOP2B (HR = 1.2; *p* = 0.16), and C1orf35 (HR = 1.2; *p* = 0.34) were not significantly correlated with DFS. Overall, the survival analyses for both OS and DFS highlight the prognostic significance of these genes, positioning them as promising biomarkers for predicting outcomes in HCC patients.

### 3.8 Hub Genes and Drug Interaction Analysis

**Figure 9:**
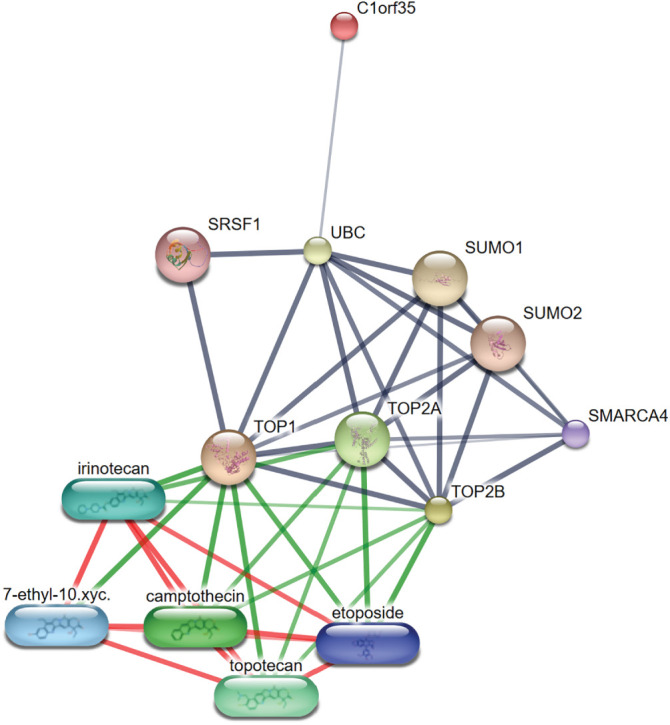
Interaction network of TOP1, TOP2A, TOP2B, and C1orf35 with regulatory proteins and chemotherapeutic agents. The network centers on TOP1, TOP2A, TOP2B, and C1orf35, illustrating their interactions with regulatory proteins such as UBC, SUMO1/2, SRSF1, and SMARCA4. Chemotherapeutic agents, including irinotecan, camptothecin, topotecan, and etoposide, are shown to interact primarily with TOP1 and TOP2 family members, emphasizing their therapeutic relevance in hepatocellular carcinoma. The connectivity of these nodes underscores the central role of topoisomerases and associated pathways in cancer-related signaling and drug response

We explored potential drug interactions targeting TOP1, TOP2A, TOP2B, and C1orf35 using the DGIdb database (https://dgidb.org) [34]. This analysis identified a total of 60 candidate drugs that may be effective in treating hepatocellular carcinoma (HCC) by modulating these four hub genes. The drug-gene interaction data were visualized and systematically screened through the DGIdb platform. To refine the selection, we focused exclusively on FDA-approved drugs, highlighting four promising agents which demonstrated shared regulatory activity associated with TOP1, TOP2A, and TOP2B. These findings provide a foundation for potential therapeutic strategies targeting critical molecular drivers in HCC. A comprehensive protein-protein and drug-target interaction network analysis revealed critical regulatory associations centered around the DNA topoisomerases TOP1, TOP2A, and TOP2B. These enzymes, essential for DNA replication and transcription, were found to interact strongly with post-translational regulators such as UBC (ubiquitin C), SUMO1, SUMO2, and SRSF1, suggesting modulation through ubiquitination and SUMOylation. Additional interactions with the chromatin remodeler SMARCA4 highlight the integration of topoisomerase function within broader epigenetic and transcriptional regulatory pathways. Drug interaction mapping demonstrated that clinically relevant chemotherapeutics, including etoposide, camptothecin, irinotecan, and topotecan, directly target TOP1 and TOP2A, supporting their established roles in anticancer strategies through inhibition of DNA topology resolution as shown in Fig. 9. The strong connectivity and diverse regulatory interactions underscore the centrality of topoisomerases in genome stability and chemotherapy response. Notably, a weaker interaction with C1orf35 suggests a potential, though less characterized, role in the regulatory landscape of these enzymes.

The network map highlights the protein-protein and drug-target interactions associated with TOP1, TOP2A, and TOP2B. Critical regulators such as UBC, SUMO1/2, SRSF1, and SMARCA4 were identified as modulators of topoisomerase activity. Chemotherapeutic agents like etoposide, camptothecin, irinotecan, and topotecan exhibited direct interactions with these targets, emphasizing their therapeutic relevance in cancer treatment, as detailed in Table 4. Importantly, no drugs were found that specifically inhibit C1orf35 based on our DGIdb database analysis. Further research is needed to better understand C1orf35 and explore potential therapeutic strategies targeting its function. Overall, this drug-gene interaction mapping highlights the clinical promise of targeting TOP1, TOP2A, and TOP2B in hepatocellular carcinoma (HCC) therapy, offering a focused selection of candidates for drug repurposing or combination therapy development.

**Table 4:** Summary of interactions between TOP1, TOP2A, TOP2B and C1orf35, regulatory proteins, and chemotherapeutic agents

Category	Interactor	Type	Primary target (s)	Interaction description
Regulatory protein	UBC	Ubiquitin-conjugating enzyme	TOP1, TOP2A, TOP2B	Involved in ubiquitin-mediated regulation
Regulatory protein	SUMO1	SUMOylation factor	TOP1, TOP2A, TOP2B	Post-translational modification via SUMOylation
Regulatory protein	SUMO2	SUMOylation factor	TOP1, TOP2A, TOP2B	Similar function to SUMO1
Regulatory protein	SRSF1	Splicing factor	TOP1	May influence transcription and mRNA processing
Regulatory protein	SMARCA4	Chromatin remodeler	TOP2A, TOP2B	Regulates access to chromatin during replication
Regulatory protein	C1orf35	Uncharacterized protein	UBC	Weak/indirect interaction
Drug	Irinotecan*	Chemotherapeutic agent	TOP1	Topoisomerase I inhibitor
Drug	Camptothecin*	Chemotherapeutic agent	TOP1	Classic TOP1 poison
Drug	Topotecan*	Chemotherapeutic agent	TOP1	TOP1 inhibitor (derivative of camptothecin)
Drug	Etoposide*	Chemotherapeutic agent	TOP2A, TOP2B	Induces DNA breaks via TOP2 inhibition
Drug	7-ethyl-10-xyc. (SN-38)	Active metabolite of irinotecan	TOP1	Potent inhibitor of TOP1

Note: *FDA-approved chemotherapeutic agents.

### 3.9 Comparative Toxicogenomics Database (CTD) Analysis

**Figure 10:**
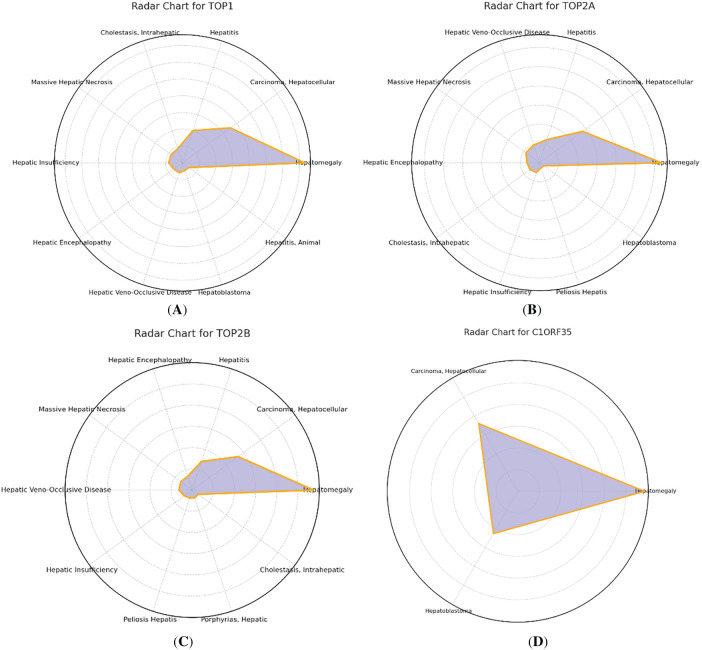
Comprehensive comparative analysis of the four genes ((**A**) TOP1, (**B**) TOP2A, (**C**) TOP2B, and (**D**) C1orf35) in relation to a spectrum of hepatic disorders, based on data from the comparative toxicogenomics database (CTD)

In this investigation, the curated list of hub genes was analyzed using the Comparative Toxicogenomics Database (CTD) to explore potential associations with various diseases, aiming to deepen our insights into gene disease interactions. The analysis identified a strong connection between the core genes TOP1, TOP2A, TOP2B, C1orf35 and a spectrum of hepatic disorders. These included Hepatocellular Carcinoma, both intrahepatic and extrahepatic cholestasis, hepatic encephalopathy, multiple forms of hepatitis (including types B and C), hepatomegaly, severe hepatic necrosis, liver insufficiency, veno-occlusive disease, autoimmune hepatitis, hepatoblastoma, and chronic hepatitis B and C as illustrated in Fig. 10. These associations emphasize the possible involvement of these genes in liver disease pathogenesis and their relevance as potential diagnostic or therapeutic targets.

### 3.10 Immunohistochemistry Analysis

**Figure 11:**
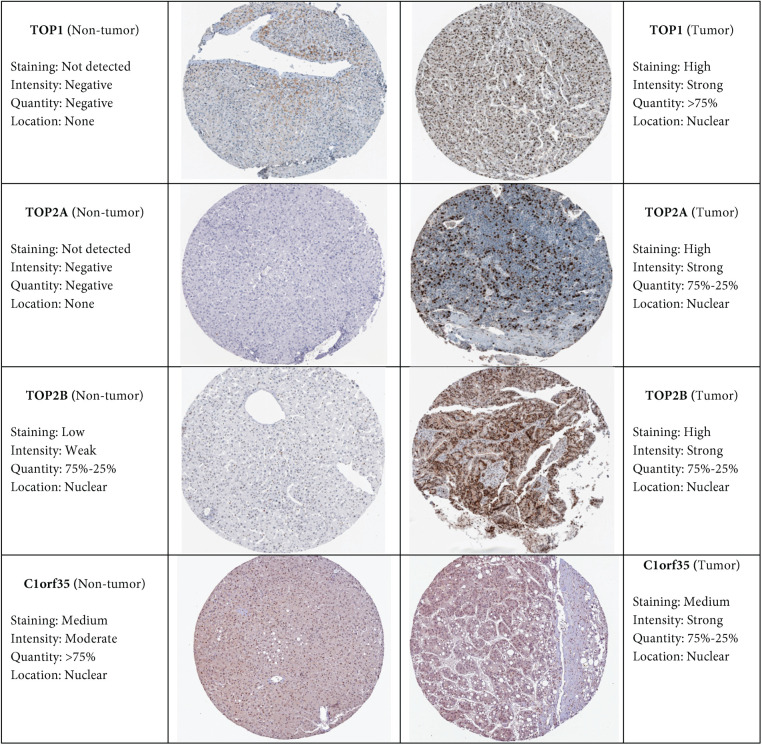
Immunohistochemistry images showing the expression of 4 hub genes in normal liver tissue and hepatocellular carcinoma (HCC) tissue, with a 50 μm scale bar. Images were obtained from the human protein atlas (HPA) database (http://www.proteinatlas.org/) [31]

The Human Protein Atlas (HPA) (http://www.proteinatlas.org) is an extensive resource that provides immunohistochemistry (IHC)-based protein expression profiles across a wide range of human tissues and cell types [31]. In this study, we utilized IHC images from the HPA to evaluate and compare the protein expression of selected target genes between normal liver tissues and hepatocellular carcinoma (HCC) tissues, as depicted in Fig. 11. Notably, TOP1, TOP2A, and TOP2B showed medium to high expression levels in HCC tissues, whereas their expression was undetectable in normal liver samples. In contrast, C1orf35 was expressed in both normal and tumor tissues, suggesting a more ubiquitous expression pattern. These observations highlight significant differences in protein expression between normal and cancerous liver tissues, underscoring the potential role of these genes in HCC development, progression, and prognostic evaluation.

### 3.11 Prediction of miRNA Targets Associated with Hub Genes

To explore the post-transcriptional regulation of the identified hub genes, we utilized the TargetScan database to predict potential miRNA interactions as shown in Table 5. The analysis revealed that TOP2A is potentially regulated by hsa-miR-144-3p at position 166–172 of TOP2A 3^′^ UTR, whereas TOP1 is targeted by hsa-miR-499a-5p at Position 104–111 of TOP1 3^′^ UTR. For TOP2B, the candidate miRNAs include hsa-miR-223-3p, which binds at position 561–568 of TOP2B 3^′^ UTR. Additionally, C1orf35 was predicted to interact with hsa-miR-216b-5p at position 1930–1936 of C1orf35 3^′^ UTR. These findings provide insights into the potential miRNA-mediated regulatory networks involved in the expression of these key genes.

**Table 5:** Predicted miRNA-mRNA interactions and their relative binding affinities (KD)

Gene	Predicted miRNA	Binding site position (3^′^ UTR)	Site type	Context++ score percentile	Predicted relative KD
TOP1	hsa-miR-499a-5p	104–111	8mer	88	−4.76
TOP2A	hsa-miR-144-3p	166–172	7mer-m8	88	−3.11
TOP2B	hsa-miR-223-3p	561–568	8mer	96	−4.93
C1orf35	hsa-miR-216b-5p	1930–1936	7mer-m8	85	−3.60

### 3.12 Genomic Alterations of TOP1, TOP2A, TOP2B, and C1orf35

**Figure 12:**
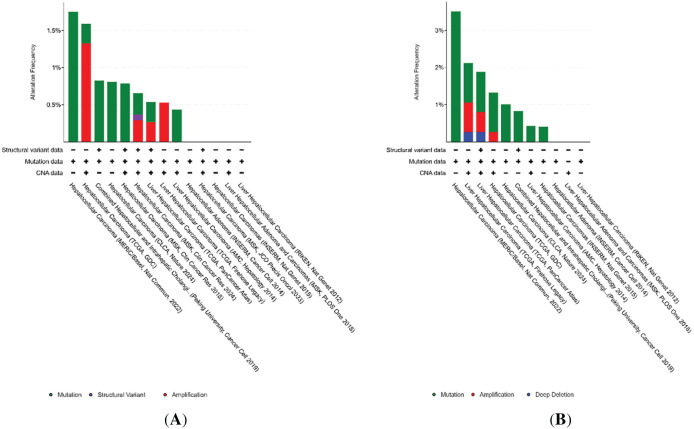

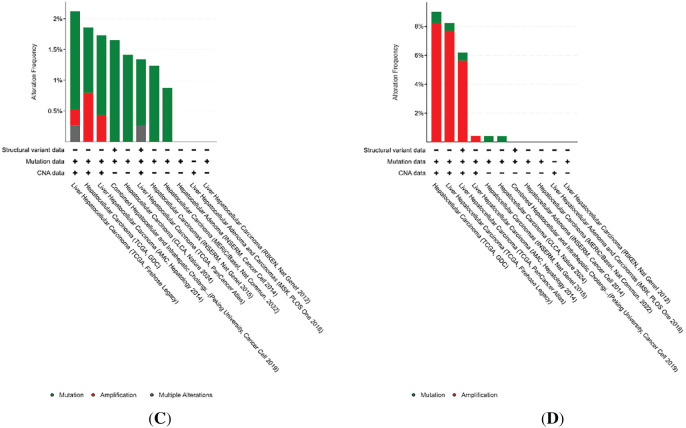
Genetic alteration frequencies of TOP1, TOP2A, TOP3A, and C1orf35 across various cancer types (14 independent datasets). (**A**) TOP1, (**B**) TOP2A, (**C**) TOP2B, and (**D**) C1orf35 alteration frequencies, including mutations, amplifications, and deep deletions, as obtained from cBioPortal. Bar colors represent different types of genomic alterations as indicated in the legend

**Figure 13:**
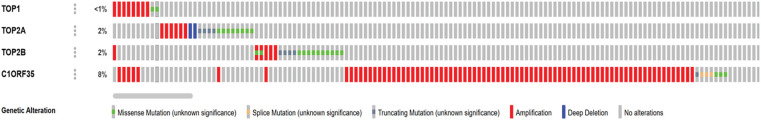
Type and frequency of TOP1, TOP2A, TOP2B, and C1orf35 gene mutations in LIHC

**Figure 14:**
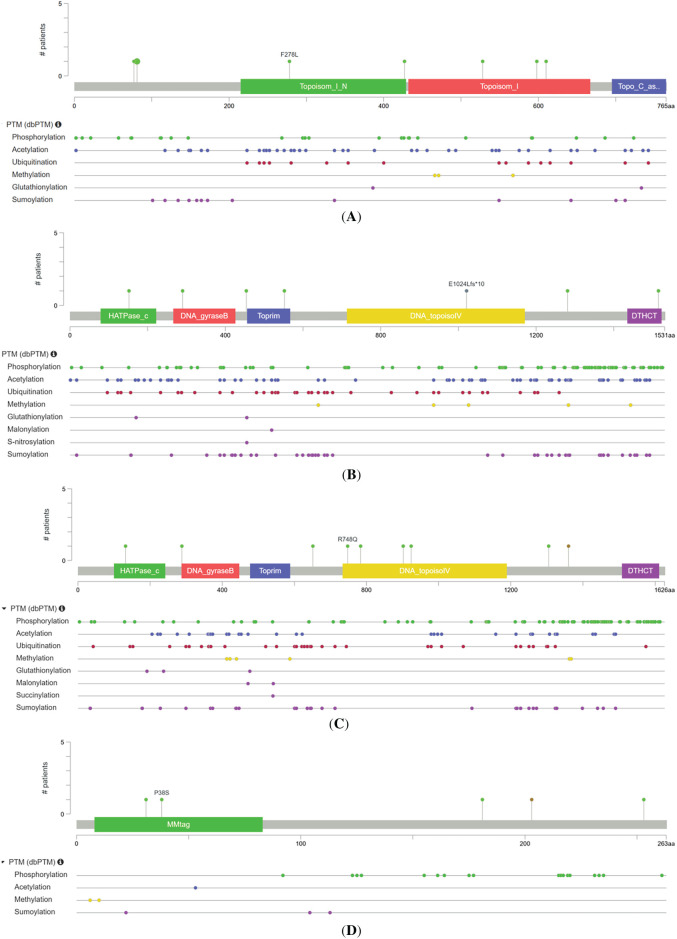
Mutation sites for TOP1, TOP2A, TOP2B, and C1orf35 mutations in the mutation frequency. (**A**) TOP1, (**B**) TOP2A, (**C**) TOP2B, and (**D**) C1orf35 showing annotated functional domains along the protein sequence and the distribution of reported PTMs, including phosphorylation, acetylation, ubiquitination, methylation, and SUMOylating. Colored symbols indicate the type and position of PTMs across the amino acid sequence

To investigate the mutational landscape of the TOP1, TOP2A, TOP2B, and C1orf35 genes in hepatocellular carcinoma (HCC), we utilized the cBioPortal platform to analyze genomic data from 14 independent datasets [39]. Fig. 12 illustrates the frequency and types of genetic alterations in these genes across multiple cancer studies sourced from The Cancer Genome Atlas (TCGA) and other major genomic projects. The *Y*-axis represents alteration frequency (%), while the *X*-axis lists the individual cancer studies. The highest alteration frequencies for TOP1 (~1.5%), TOP2A (~3%), and TOP2B (~2%) were observed in HCC, with somatic mutations being the predominant alteration type. These findings suggest a potential role for these genes in the molecular pathogenesis of HCC. Notably, C1orf35 (~8%) exhibited the highest alteration frequency among the four genes, primarily due to gene amplification as illustrated in Fig. 13. This trend was also observed in several other cancer types, indicating a possible oncogenic role for C1orf35 driven predominantly by copy number gains. This comprehensive genomic profiling underscores the potential utility of TOP1, TOP2A, TOP2B, and C1orf35 as biomarkers or therapeutic targets in HCC and potentially other tumor types. Our mutational analysis identified distinct mutation sites within TOP1, TOP2A, TOP2B, and C1orf35, as presented in Fig. 14. Further analysis of TCGA data revealed that copy number alterations (CNAs) involving these genes were among the most prevalent genomic events in HCC samples, with mutation-related CNA frequencies estimated between 0.5% and 8%.

Further analysis of 372 cases from the TCGA PanCancer Atlas, 379 cases from TCGA GDC, and 379 cases from the TCGA Firehose Legacy revealed a significant association between copy number alterations (CNAs) in *TOP1, TOP2A, TOP2B*, and *C1orf35* and their respective expression levels in liver cancer tissues. Additionally, we analyzed genomic alterations in 1130 samples from 1126 liver hepatocellular carcinoma (LIHC) patients across three TCGA studies as shown in Fig. 14 and Supplementary Table S6 (Type of Genetic alterations). We identified two cases of TOP1 mutations (0.2%), including one duplicate missense mutation (*E695K*) found in patients with multiple samples. A total of 12 mutations (1.1%) involving TOP2A were observed, including five duplicate mutations across multiple samples. These included eight missense mutations (*I70T, T689N, R877W, T1315K*, and *T1441N*) and four truncating mutations, with one notable frameshift deletion (*K1199Rfs*25*). Moreover, TOP2B exhibited 17 mutations, including four duplicates in patients with multiple samples. These mutations consisted of 13 missense variants (*K1256R, K1255E, K1250E, P886S, P881S, H699Q, E76Q, E71Q*, and *E62K*) and four frameshift deletions (W619*, W614*, *V174Lfs*22*). In the case of C1orf35, seven mutations were identified, including four duplicates across multiple samples. These comprised three missense mutations (*G157E*), one frameshift deletion (*F148Sfs*6*), and three splice site mutations (*X82_splice*). Notably, there is currently no information available about genes in the OncoKB database. This analysis highlights the differential alteration burden across these genes, suggesting a more prominent role for *C1orf35* and *TOP2A* in HCC through genomic amplification and mutation events. These findings are consistent with broader analyses from TCGA and cBioPortal, supporting their potential as candidate therapeutic targets or prognostic biomarkers.

### 3.13 The Relationship between TOP1, TOP2A, TOP2B, C1orf35 Expressions and Hepatocellular Carcinoma Immune Cell Immersion

**Figure 15:**
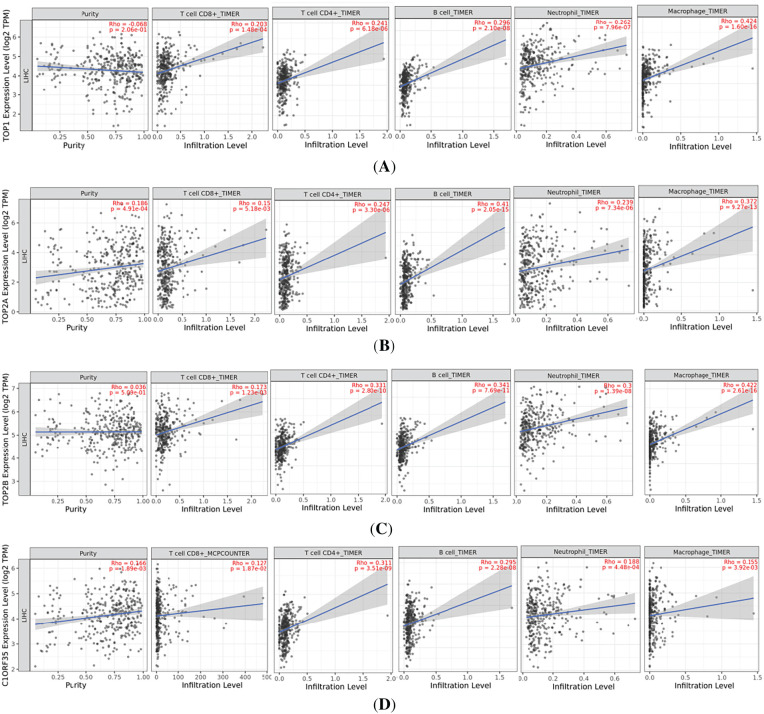
Correlation between (**A**) TOP1, (**B**) TOP2A, (**C**) TOP2B, and (**D**) C1orf35 expression levels and hepatocellular carcinoma immune cell infiltration levels; Rho = the Spearman’s correlation coefficient; Blue line: the regression line showing the general trend; Gray shaded area: confidence interval around the regression line, indicating variability and uncertainty in the estimate.

To investigate the immunological significance of TOP1, TOP2A, TOP2B, and C1orf35 in hepatocellular carcinoma (HCC), we examined their correlation with tumor infiltrating immune cells using the TIMER database, [40] as illustrated in Fig. 15. Scatter plots generated through TIMER depict the association between TOP1 expression levels (log_2_ TPM) and tumor purity, as well as the infiltration levels of various immune cell subsets, including CD8^+^ T cells, CD4^+^ T cells, B cells, neutrophils, and macrophages, in liver hepatocellular carcinoma (LIHC) samples. No statistically significant correlation was observed between TOP1 expression and tumor purity (Rho = −0.068, *p* = 0.206). In contrast, TOP1 expression demonstrated significant positive correlations with infiltration levels of CD8^+^ T cells (Rho = 0.203, *p* = 1.48 × 10^−4^), CD4^+^ T cells (Rho = 0.241, *p* = 6.18 × 10^−6^), B cells (Rho = 0.296, *p* = 2.10 × 10^−8^), neutrophils (Rho = 0.262, *p* = 7.96 × 10^−7^), and macrophages (Rho = 0.424, *p* = 1.6 × 10^−16^). These findings suggest that elevated TOP1 expression may be associated with increased immune cell infiltration, particularly of macrophages and B cells, within the tumor microenvironment. This association indicates a potential immunomodulatory function of TOP1 in HCC and highlights its possible role in shaping tumor immune interactions during liver cancer progression.

To evaluate the potential immunological relevance of TOP2A in hepatocellular carcinoma (HCC), we analyzed its expression in relation to tumor purity and immune cell infiltration using the TIMER database. A statistically significant positive correlation between TOP2A expression and tumor purity (Rho = 0.186, *p* = 4.91 × 10^−4^), suggesting that elevated TOP2A expression is more prominent in tumor enriched tissue samples, potentially reflecting its role in tumor cell proliferation or genomic instability. In addition, significant positive correlations with infiltration levels of CD8^+^ T cells (Rho = 0.15, *p* = 5.18 × 10^−3^), CD4^+^ T cells (Rho = 0.247, *p* = 3.30 × 10^−6^), B cells (Rho = 0.41, *p* = 2.05 × 10^−15^), neutrophils (Rho = 0.239, *p* = 7.34 × 10^−6^), and macrophages (Rho = 0.372, *p* = 9.27 × 10^−13^). Collectively, these findings suggest that TOP2A may contribute to both tumor biology and immune landscape modulation in HCC, supporting its potential utility as a biomarker or therapeutic target in liver cancer. Moreover, to explore the immunological significance of TOP2B in hepatocellular carcinoma (HCC), we analyzed its association with tumor infiltrating immune cells. No statistically significant correlation was observed between TOP2B expression and tumor purity (Rho = 0.036, *p* = 0.509). In contrast, the scatter plot analysis revealed statistically significant positive correlations between TOP2B expression and the infiltration levels of CD8^+^ T cells, CD4^+^ T cells, B cells, neutrophils, and macrophages in (LIHC) samples. Specifically, significant positive correlation was observed with CD8^+^ T cells (Rho = 0.173, *p* = 1.23 × 10^−3^), CD4^+^ T cells (Rho = 0.331, *p* = 2.80 × 10^−10^), B cells (Rho = 0.341, *p* = 7.69 × 10^−11^), Neutrophils (Rho = 0.300, *p* = 1.39 × 10^−8^), and Macrophages (Rho = 0.422, *p* = 2.61 × 10^−16^). These findings highlight a compelling relationship between elevated TOP2B expression and enhanced immune cell infiltration, underscoring the potential immunomodulatory role of TOP2A in shaping the tumor microenvironment.

To assess the immunological relevance of C1orf35 in hepatocellular carcinoma (HCC), A significant positive correlation was observed between C1orf35 expression and tumor purity (Rho = 0.166, *p* = 1.89 × 10^−3^), indicating that higher levels of C1orf35 are predominantly found in tumor rich tissue, which may reflect its involvement in cellular proliferation and genomic instability. Moreover, C1orf35 expression exhibited significant positive associations with the infiltration of several immune cell populations, including; CD8^+^ T cells (Rho = 0.127, *p* = 1.87 × 10^−2^), CD4^+^ T cells (Rho = 0.311, *p* = 3.51 × 10^−9^), B cells (Rho = 0.295, *p* = 2.28 × 10^−8^), Neutrophils (Rho = 0.188, *p* = 4.48 × 10^−4^), and Macrophages (Rho = 0.155, *p* = 3.92 × 10^−3^). These findings collectively imply that C1orf35 not only plays a role in tumor progression but also may influence the immune landscape of the HCC microenvironment. Thus, TOP2A emerges as a promising candidate for further investigation as both a biomarker and a potential immunotherapeutic target in liver cancer.

### 3.14 TOP1, TOP2A, TOP2B, and C1orf35 Expression in Cancer Tissues

**Figure 16:**
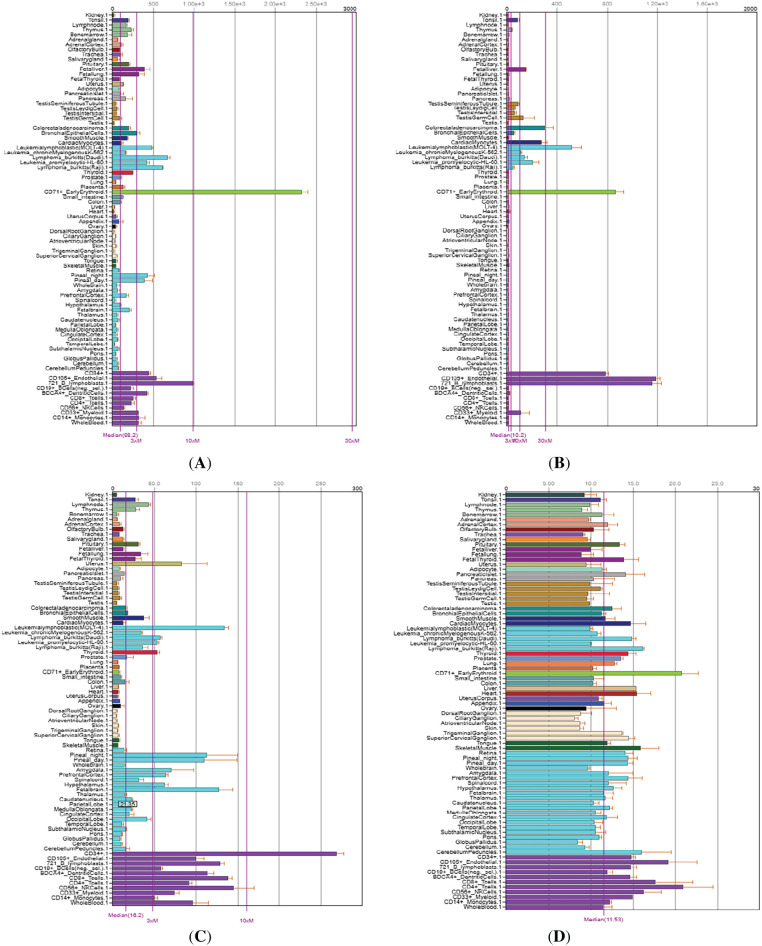
Comparative expression analysis of TOP1, TOP2A, TOP2B, and C1orf35 across multiple cancer types using BioGPS database. (**A**) TOP1, (**B**) TOP2A, (**C**) TOP2B, and (**D**) C1orf35 showing the positional distribution and frequency of reported post-translational modifications along the protein sequence. Bars represent the relative abundance of modification sites, with different colors indicating distinct modification types as defined in the legend

An online analysis using the BioGPS database reveals that the TOP1, TOP2A, TOP2B, and C1orf35 genes are broadly expressed across most normal human tissues. Notably, TOP1 expression in liver tissue is slightly below the average expression level of 98.2, with a reported median value of 25.2. TOP2A expression in liver tissue is slightly above the average level of 10.2, with a reported median value of 16.65. TOP2B expression in liver tissue is slightly below the average level of 16.2, with a reported median value of 7.05. In contrast, C1orf35 expression in liver tissue is slightly above the average level of 11.3, with a reported median value of 15.4, as presented in the Fig. 16. Data compiled from multiple databases consistently indicate that TOP2A and C1orf35 expression is significantly elevated in cancerous tissues compared to normal tissues. To reinforce the reliability of this observation, we cross-validated the expression patterns of TOP1, TOP2A, TOP2B, and C1orf35 using independent datasets from distinct repositories. By leveraging these diverse data sources, we enhanced the validity and robustness of our findings, minimizing potential bias and increasing the credibility of our conclusions.

## 4 Discussion

Hepatocellular carcinoma (HCC) ranks among the most prevalent malignancies globally. Although notable progress has been made in therapeutic strategies over the last twenty years—including surgical removal, local ablative procedures, liver transplantation, and molecularly targeted treatments—long-term patient outcomes remain unsatisfactory, with low five-year survival rates [44]. The use of radiotherapy is limited because of the high sensitivity of healthy liver tissue to radiation-induced damage. In addition, liver transplantation faces major challenges, such as organ scarcity, restricted eligibility outside the Milan criteria, and substantial recurrence rates, particularly in cases linked to hepatitis C virus (HCV) infection [45]. HCC is characterized by high mortality rates, underscoring the urgent need for effective biomarkers to guide treatment strategies. Posttranslational modifications (PTMs), such as phosphorylation and dephosphorylation, play crucial roles in regulating protein functions and interactions [1]. These reversible modifications influence more than 70% of cellular proteins, shaping key intracellular signaling pathways. While kinases enzymes responsible for phosphorylation are well-characterized, phosphatases, which mediate dephosphorylation, remain less studied. Although the human genome contains around 518 protein kinases, it encodes only about 137 phosphatases, revealing a substantial disparity and underscoring the limited understanding of phosphatase biology compared to kinases [6]. Given the complex molecular mechanisms underlying HCC, there is a pressing need for prognostic biomarkers to improve patient outcomes and facilitate personalized treatment strategies. While recent advancements in gene sequencing technology have led to the discovery of predictive gene markers, their numbers remain limited [46]. To address this, our study leverages Comprehensive Proteomic Analysis through CPTAC to identify novel biomarkers with improved predictive accuracy, aiming to enhance HCC prognosis and patient care.

Our initial analysis identified 11,547 phosphorylation sites associated with 4043 phosphoproteins, with these sites being quantified in over 50% of the samples. Among them, 9994 phosphorylation sites exhibited significant differential expression, with 8198 sites showing upregulation and 1796 sites showing downregulation in tumor samples compared to their non-tumor counterparts. These differences were determined using a paired two-sided Student’s *t*-test, with Benjamini-Hochberg (BH)-adjusted *p*-values **<** 0.05. From this dataset, we focused on 14 phosphorylation sites that demonstrated significant changes between tumor and non-tumor samples (*p*-values < 0.0001). Notably, our comprehensive analysis of 4 phosphoproteins and their associated phosphorylation sites revealed a upregulation pattern in 4 unique genes. These results highlight the importance of continued research into the functional significance of these proteins and their associated phosphorylation sites in hepatocellular carcinoma (HCC). In this work, we performed a detailed analysis of genes showing increased expression and their corresponding phosphosites, with particular emphasis on TOP1, TOP2A, TOP2B, and C1orf35. Advanced bioinformatics approaches were applied to quantify expression patterns and to pinpoint candidates with potential therapeutic relevance. The primary objective was to evaluate the clinical implications of these genes and clarify their contribution to HCC progression. Through the use of targeted phosphopeptide enrichment, this study reveals previously unreported phosphoproteomic features associated with liver cancer, enhancing our understanding of phosphorylation-driven signaling networks in tumor-derived cell lines.

Through an integrated analysis of gene expression, phosphorylation data, miRNA, CTD, TIMER, and patient survival data, four genes (TOP1, TOP2A, TOP2B, and C1orf35) were identified as critical candidates potentially involved in the progression of hepatocellular carcinoma (HCC). Currently, little studies have shown that these core genes may act as carcinogenic genes or promising biomarkers of cancer. In this study, we examine the post-translational modifications (PTMs) that influence TOP1, TOP2A, TOP2B, and C1orf35 in both normal and cancerous cells, emphasizing the structural and functional implications of these modifications. These genes play a crucial role in managing DNA topology during replication, transcription, and chromosome segregation, making it a significant target in cancer chemotherapy. PTMs such as phosphorylation, acetylation, ubiquitination, SUMOylation, and O-GlcNAcylation have been identified as key regulators of TOP2A’s activity, stability, and subcellular localization. Moreover, We quantified significant hyperphosphorylation sites on TOP1 at S97 (Log_2_FC = 2.2042; adjusted *p*-value = 1.68 × 10^−32^); on TOP2A at S1377 (Log_2_FC = 2.8393; adjusted *p*-value = 1.93 × 10^−58^), S1106 (Log_2_FC = 2.6018; adjusted *p*-value = 1.17 × 10^−61^), S1351 (Log_2_FC = 2.2055; adjusted *p*-value = 7.05 × 10^−44^), S1354 (Log_2_FC = 2.0393; adjusted *p*-value = 1.28 × 10^−27^), S1374 (Log_2_FC = 2.0386; adjusted *p*-value = 3.39 × 10^−37^), S1213 (Log_2_FC = 1.5915; adjusted *p*-value = 5.66 × 10^−39^), and S1295 (Log_2_FC = 1.1924; adjusted *p*-value = 1.03 × 10^−30^); on TOP2B at S1408 (Log_2_FC = 1.2646; adjusted *p*-value = 1.13 × 10^−27^) and S1547 (Log_2_FC = 0.9550; adjusted *p*-value = 4.25 × 10^−39^); and on C1orf35 at S231 (Log_2_FC = 2.9102; adjusted *p*-value = 1.45 × 10^−22^), S233 (Log_2_FC = 2.9100; adjusted *p*-value = 1.45 × 10^−22^), S177 (Log_2_FC = 1.5096; adjusted *p*-value = 1.00 × 10^−30^), S217 (Log_2_FC = 1.4009; adjusted *p*-value = 2.87 × 10^−33^), and T175 (Log_2_FC = 1.2385; adjusted *p*-value = 1.07 × 10^−25^) in tumor patients compared to non-tumor patients. Our study offers a comprehensive exploration of the phosphoproteomic landscape in hepatocellular carcinoma (HCC), unveiling novel insights into disease mechanisms and identifying potential biomarkers for improved diagnostic and prognostic applications. Through high-resolution phosphoproteomic profiling, we characterized distinct phosphorylation patterns associated with HCC progression. These patterns revealed significant alterations in signaling pathways related to cell cycle regulation, DNA repair, and immune response, underscoring the complexity of HCC pathogenesis.

DNA topoisomerases are essential enzymes that manage the topological states of DNA, ensuring proper cellular function during processes like replication and transcription. In eukaryotic cells, the primary topoisomerases include TOP1 and TOP2A [47]. The C-terminal domain (CTD) of human DNA topoisomerase IIα (TOP2A) is a focal point for phosphorylation events that modulate the enzyme’s localization and activity in both normal and cancerous cells. Phosphorylation at specific serine residues within the CTD plays critical roles in regulating TOP2A function [48]. Phosphorylation of serine 1213 (Ser1213) is particularly important for the enzyme’s relocalization during mitosis. Ser1213-phosphorylated TOP2A first appears on chromosome arms in prophase, becomes concentrated at centromeres during metaphase, and disappears in early telophase, indicating a role in chromosome condensation and segregation. Casein kinase I delta and epsilon (CKIδ/ε) phosphorylate serine 1106 (Ser1106), enhancing TOP2A’s DNA cleavage activity [48]. Mutation of Ser1106 to alanine results in decreased enzyme function, underscoring the importance of this modification. Conversely, not all phosphorylation events directly influence TOP2A activity. For instance, substitution of serine 1525 (Ser1525) with alanine does not affect decatenation activity, despite Ser1525 being a major substrate for phosphorylation by CKII, Polo-like kinase I, and p38γ [49].

To uncover overrepresented cancer hallmark, functional enrichment analysis using the MSigDB (Molecular signatures database) Hallmark gene sets revealed significant associations between the genes of interest and multiple cancer-related pathways [50]. Notably, TOP2A was enriched in several key biological processes, including apoptosis, E2F targets, estrogen response (late), G2/M checkpoint, mitotic spindle, and peroxisome activity, with adjusted *p*-values below 0.05, indicating statistical significance. The most pronounced enrichment was observed in the G2/M checkpoint pathway, where both TOP1 and TOP2A were involved, yielding an adjusted *p*-Value of 0.0036, an odds ratio of 124.26, and the highest combined score of 938.19. This suggests a strong link between these genes and cell cycle regulation. Additional enrichment of TOP1 was observed in the heme metabolism pathway, further supporting its functional involvement in cancer-related biological mechanisms. The results underscore the pivotal roles of TOP1 and TOP2A in pathways that are critical to tumor progression and cell proliferation as illustrated in Table 6. No enrichment data were available for C1orf35, highlighting the need for further investigation. A deeper understanding of such hallmarks can significantly benefit cancer prevention, diagnosis, and treatment development by summarizing the functional and metabolic commonalities underlying malignant transformation and progression.

**Table 6:** The hallmarks of cancer framework offers a comprehensive basis for understanding the fundamental biological principles shared across diverse types of cancers

Term	Overlap	*p-*value	Adjusted *p-*value	Odds ratio	Combined score	Genes
HALLMARK_APOPTOSIS	1/161	0.032	0.039	55.467	191.167	TOP2A
HALLMARK_E2F_TARGETS	1/200	0.039	0.039	44.507	143.854	TOP2A
HALLMARK_ESTROGEN _RESPONSE_LATE	1/200	0.039	0.039	44.507	143.854	TOP2A
HALLMARK_G2M _CHECKPOINT	2/200	0.001	0.004	124.26	938.190	TOP1; TOP2A
HALLMARK_HEME _METABOLISM	1/200	0.039	0.039	44.507	143.854	TOP1
HALLMARK_MITOTIC _SPINDLE	1/199	0.039	0.039	44.734	144.809	TOP2A
HALLMARK_PEROXISOME	1/104	0.021	0.039	86.345	334.990	TOP2A

Maintaining a precise balance between phosphorylation and dephosphorylation is essential for proper cellular function. Disruptions in this equilibrium, governed by protein kinases and phosphatases, can lead to various diseases, including cancer [1]. In hepatocellular carcinoma (HCC), identifying early biomarkers remains challenging due to the complex molecular alterations involved. Advanced proteomic and genomic techniques have facilitated the discovery of potential biomarkers. Our study identified TOP1, TOP2A, TOP2B, and C1orf35 as key genes influencing the liver microenvironment and contributing to HCC development. These genes affect signaling pathways within liver cells, thereby impacting HCC progression, and highlighting their potential as therapeutic targets. Furthermore, we conducted a comprehensive analysis of KEGG pathways and Gene Ontology (GO) terms associated with the phosphorylated proteins to further understand their roles in HCC pathogenesis.

Gene Ontology (GO) enrichment analysis of phosphoproteomic data has revealed significant involvement of phosphorylated proteins in critical biological processes, molecular functions, and cellular components, as well as associations with key pathways and diseases. In the biological process category, there is notable enrichment in DNA repair mechanisms, including T-circle formation and DNA metabolic processes, highlighting the role of these proteins in maintaining genomic integrity. Additionally, these proteins are implicated in the cellular response to DNA damage stimuli and chromosome segregation, underscoring their importance in cell cycle regulation and genomic stability. Molecular function analysis indicates significant enrichment in catalytic activities acting on DNA, such as ATP-dependent activities, DNA topoisomerase activity, and nuclease activity. These functions are essential for DNA replication, repair, and transcription. Furthermore, activities like single-stranded DNA binding and forked DNA-dependent helicase activity suggest roles in DNA unwinding and processing during replication and repair processes. Regarding cellular components, the phosphorylated proteins are predominantly localized within the chromosome, nucleoplasm, nucleolus, nuclear body, and intracellular organelle lumen. This distribution indicates their central role in nuclear processes and the maintenance of nuclear architecture.

Pathway enrichment analysis using KEGG has identified significant involvement in the Homologous Recombination and Fanconi Anemia pathways, both crucial for the repair of DNA double-strand breaks and interstrand cross-links, thereby maintaining genomic stability. Complementary analysis using Wiki Pathways has highlighted key DNA damage response mechanisms, including the DNA IR-damage and cellular response via ATR and DNA IR-double strand breaks and cellular response via ATM pathways, emphasizing the role of these proteins in responding to DNA damage. Further functional enrichment through Gene Set Enrichment Analysis (GSEA) of Reactome pathways has underscored the involvement of these phosphoproteins in critical biological processes such as DNA Double-Strand Break Repair, Homology-Directed Repair through Homologous Recombination, SUMOylation, G2/M DNA damage checkpoint, cell cycle regulation, and post-translational protein modifications, including the regulation of TP53 activity through phosphorylation and impaired BRCA2 binding to RAD51. Moreover, disease-gene association analysis for the upregulated genes TOP1, TOP2A, TOP2B, and C1orf35 has revealed strong links to genetic disorders such as Bloom syndrome, Werner syndrome, Rothmund-Thomson syndrome, Spinocerebellar ataxia type 1 with axonal neuropathy, and liver cirrhosis. These associations underscore the clinical relevance of these phosphoproteins in various genetic diseases and their potential as therapeutic targets. This comprehensive analysis provides valuable insights into the functional roles of phosphorylated proteins in DNA repair, cellular responses to DNA damage, and their implications in disease pathogenesis. The biological function of C1orf35 remains largely uncharacterized. However, hypotheses based on co-expression patterns, enrichment analyses, and PPI predictions suggest that C1orf35 may be involved in transcriptional regulatory pathways, including potential links to c-MYC regulatory networks.

Our study highlights RAD51 as a key contributor to hepatocellular carcinoma (HCC) progression. RAD51 was significantly upregulated in HCC compared with normal liver tissue, correlating with poorer overall survival and indicating its potential as a marker of tumor aggressiveness. Co-expressed genes were enriched in cell-cycle regulation, DNA repair, and genomic instability, suggesting a role for RAD51 in malignant transformation, therapeutic resistance, and maintenance of stem-like tumor subpopulations. Immune profiling revealed that RAD51 expression varied across immune subtypes and correlated with immunomodulators and immune infiltration. Proliferating T cells showed the highest RAD51 expression, suggesting potential impacts on T-cell activation and dysfunction. RAD51 also showed moderate predictive value for immune checkpoint therapy response, and TIDE analysis indicated differences in immune dysfunction and exclusion between RAD51 high and low groups [51]. Moreover, RAD51 expression negatively correlated with sensitivity to multiple chemotherapeutic and targeted agents, underscoring its role in treatment resistance. The RAD51-based prognostic nomogram demonstrated high predictive accuracy, supporting its potential as a clinical risk-stratification tool. While these findings establish RAD51 as a promising diagnostic, prognostic, and therapeutic biomarker in HCC, experimental validation through *in-vitro* and *in-vivo* studies is needed to confirm its mechanistic roles and therapeutic potential.

The interaction network illustrates the relationships between proteins and drug targets involving TOP1, TOP2A, and TOP2B. Key regulatory proteins such as UBC, SUMO1/2, SRSF1, and SMARCA4 were identified as significant modulators of topoisomerase activity. Several anticancer agents, including etoposide, camptothecin, irinotecan, and topotecan, were found to directly engage with these topoisomerases, underlining their importance in cancer therapeutics. Notably, analysis using the DGIdb database revealed no existing drugs that specifically target C1orf35. This highlights the need for additional studies to clarify the role of C1orf35 and to investigate potential avenues for therapeutic intervention. Collectively, these drug-gene interaction insights support the clinical potential of targeting TOP1, TOP2A, and TOP2B in HCC, pointing to promising opportunities for drug repositioning and combination treatment strategies.

**Figure 17:**
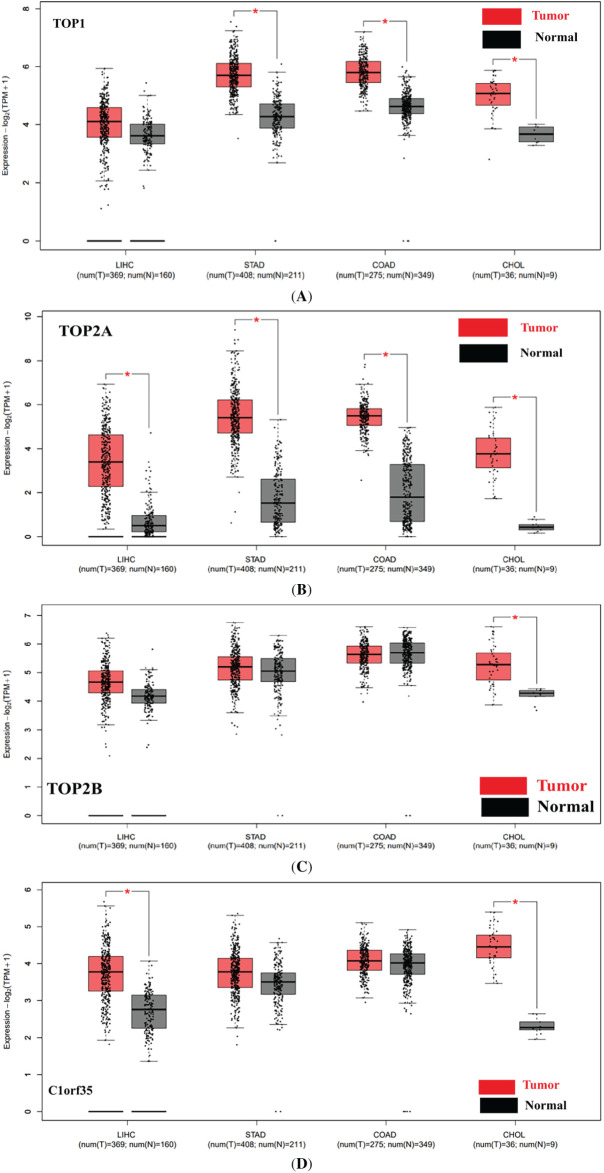
Expression levels of (**A**) TOP1, (**B**) TOP2A, (**C**) TOP2B, and (**D**) C1orf35 in hepatocellular carcinoma (HCC), comparing normal liver tissues (shown in black boxes) with HCC tissues (shown in red boxes). Data were obtained from the GEPIA online tool (*p < 0.01; tumor vs. normal)

To gain deeper insights into the functional significance of selected genes in HCC, we carried out a comprehensive expression profiling analysis using datasets from The Cancer Genome Atlas (TCGA) [52]. Our investigation centered on four genes strongly implicated in HCC, with a comparative approach involving their expression across Stomach Adenocarcinoma (STAD), Colon Adenocarcinoma (COAD), Liver Hepatocellular Carcinoma (LIHC), and Cholangiocarcinoma (CHOL). These cancer types were specifically chosen due to their known potential for hepatic metastasis. By exploring differential gene expression among these malignancies, we aimed to identify molecular signatures capable of distinguishing primary liver cancer from metastatic lesions in hepatic tissue. Gene expression analysis was performed using the GEPIA2 platform, with results visualized in Fig. 17. Differential expression was statistically assessed using Student’s *t*-test, applying cutoffs of *p*-value < 0.001 and a minimum twofold change. Our data revealed a consistent trend of elevated gene expression in tumor tissues relative to their normal counterparts. In STAD samples, TOP1 and TOP2A/B showed marked upregulation in tumors. Similarly, COAD specimens demonstrated significantly increased expression of TOP1 and TOP2A in cancerous tissues. For LIHC, both TOP2A and C1orf35 exhibited pronounced overexpression in tumor samples compared to adjacent normal tissues. Additionally, TOP1, TOP2A, TOP2B, and C1orf35 were significantly upregulated in tumor tissues. Collectively, these findings point to TOP1, TOP2A, and C1orf35 as potential diagnostic indicators for liver-related malignancies. Their consistently higher expression across all four cancer types reinforces their utility in differentiating primary hepatic tumors from metastatic counterparts and suggests their relevance as biomarkers for early cancer detection and diagnosis.

In our comprehensive analysis of hepatocellular carcinoma (HCC) patients, we observed no statistically significant difference in overall survival (OS) between groups (*p* = 0.56). However, multivariable Cox regression analysis identified several significant predictors of recurrence following liver resection. Established clinical indicators, including tumor size, number, and vascular invasion, were validated as predictors of poor outcomes. Notably, elevated expression levels of TOP1, TOP2A, TOP2B, and C1orf35 were associated with poorer OS compared to lower expression levels. Further survival analysis demonstrated that high expression of TOP1, TOP2A, TOP2B, and C1orf35 is significantly associated with poorer OS in this patient cohort. These findings suggest that these genes may serve as negative prognostic biomarkers, indicating worse outcomes when highly expressed. In disease-free survival (DFS) analysis, lower expression levels of TOP1, TOP2A, TOP2B, and C1orf35 were associated with improved DFS outcomes (*p* < 0.05). Specifically, TOP2A exhibited a strong association with improved DFS (HR = 1.7; *p* = 0.00059). In contrast, lower expression levels of TOP1 (HR = 1.1; *p* = 0.4), TOP2B (HR = 1.2; *p* = 0.16), and C1orf35 (HR = 1.2; *p* = 0.34) were insignificantly correlated with DFS. Overall, the survival analyses for both OS and DFS highlight the prognostic significance of these genes, positioning them as promising biomarkers for predicting outcomes in HCC patients.

Utilizing the STRING database, we constructed a protein-protein interaction (PPI) network and identified four central hub genes: TOP1, TOP2A, TOP2B, and C1orf35. These genes exhibited significant relevance in hepatocellular carcinoma (HCC), with higher expression levels correlating strongly with increased patient survival. This association was validated through survival analyses and assessments of gene and protein expression levels. To enhance the robustness of our findings, we validated gene expression differences between tumor and normal tissues using data from The Cancer Genome Atlas (TCGA). Additionally, immunohistochemical analyses confirmed the differential protein expression patterns of these genes in HCC samples. We also explored potential therapeutic agents targeting these genes, integrating data from the Clinical Proteomic Tumor Analysis Consortium (CPTAC), TCGA, and RTCGA databases. This comprehensive approach underscores the potential of TOP1, TOP2A, TOP2B, and C1orf35 as prognostic biomarkers and therapeutic targets in HCC.

**Figure 18:**
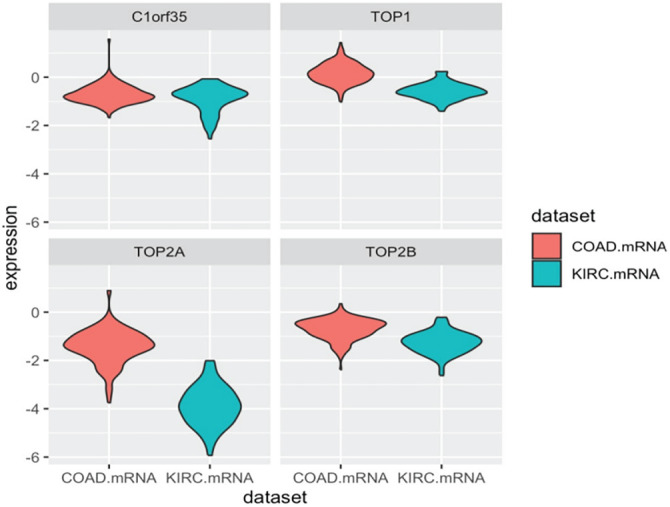
Comparative mRNA expression of four selected genes in HCC vs. COAD and KIRC datasets, based on RTCGA analysis performed using RStudio

The Cancer Genome Atlas (TCGA) is an extensive repository that offers comprehensive genomic and clinical data across various cancer types, including over 2000 primary tumors and matched normal samples [52]. For our study, we leveraged the RTCGA package within the RStudio environment to access and analyze mRNA expression profiles and associated clinical information from TCGA datasets, specifically focusing on Colon Adenocarcinoma (COAD) and Kidney Renal Clear Cell Carcinoma (KIRC). The RTCGA package facilitates the retrieval and integration of TCGA data, streamlining the process of data acquisition and analysis [53]. Our analysis aimed to compare gene expression patterns across different cancer types, with a particular focus on hepatocellular carcinoma (HCC). By examining the expression profiles of specific genes in HCC relative to COAD and KIRC, we sought to identify unique molecular signatures that could serve as potential biomarkers or therapeutic targets. The results, detailed in Fig. 18 and Supplementary Table S7 (The mRNA Expression), revealed distinct expression patterns of key genes in HCC compared to the other malignancies. These findings underscore the critical role of these genes in liver cancer pathogenesis. As our understanding of the complex molecular mechanisms driving HCC progression deepens, such insights hold promise for the development of targeted therapies aimed at modulating the activity of these genes and their associated pathways. Continued research in this area is essential to translate these molecular discoveries into clinical applications that can improve diagnosis, prognosis, and treatment outcomes for HCC patients.

We analyzed the expression profiles of TOP1, TOP2A, TOP2B, and C1ORF35 across a variety of tumor types and corresponding normal tissues based on TCGA datasets [52]. Fig. 18 presents the expression levels of TOP1 across a broad range of tumor and normal tissue samples from TCGA datasets. A significant upregulation of TOP1 was observed in several cancers, including bladder urothelial carcinoma (BLCA), colon adenocarcinoma (COAD), esophageal carcinoma (ESCA), head and neck squamous cell carcinoma (HNSC), liver hepatocellular carcinoma (LIHC), lung adenocarcinoma (LUAD), and stomach adenocarcinoma (STAD) when compared with matched normal tissues (*p* < 0.001). Notably, LIHC and STAD demonstrated particularly elevated TOP1 expression, suggesting a potential role for TOP1 in tumorigenesis and progression in these cancers. These findings support the relevance of TOP1 as a potential biomarker and therapeutic target in cancer. The expression pattern of TOP2A was analyzed across various tumor and normal tissues using TCGA datasets. As shown in Fig. 18, Significant upregulation of TOP2A was observed in several malignancies, including but not limited to bladder urothelial carcinoma (BLCA), breast invasive carcinoma (BRCA), colon adenocarcinoma (COAD), head and neck squamous cell carcinoma (HNSC), liver hepatocellular carcinoma (LIHC), lung adenocarcinoma (LUAD), and rectum adenocarcinoma (READ). Particularly, LIHC and LUAD tumors exhibited markedly higher TOP2A expression relative to their normal counterparts, highlighting the potential oncogenic role of TOP2A in hepatocellular and lung carcinogenesis. These findings underscore the frequent dysregulation of TOP2A in human cancers and suggest its relevance as a diagnostic biomarker, a prognostic indicator, and a candidate for targeted therapeutic intervention.

**Figure 19:**
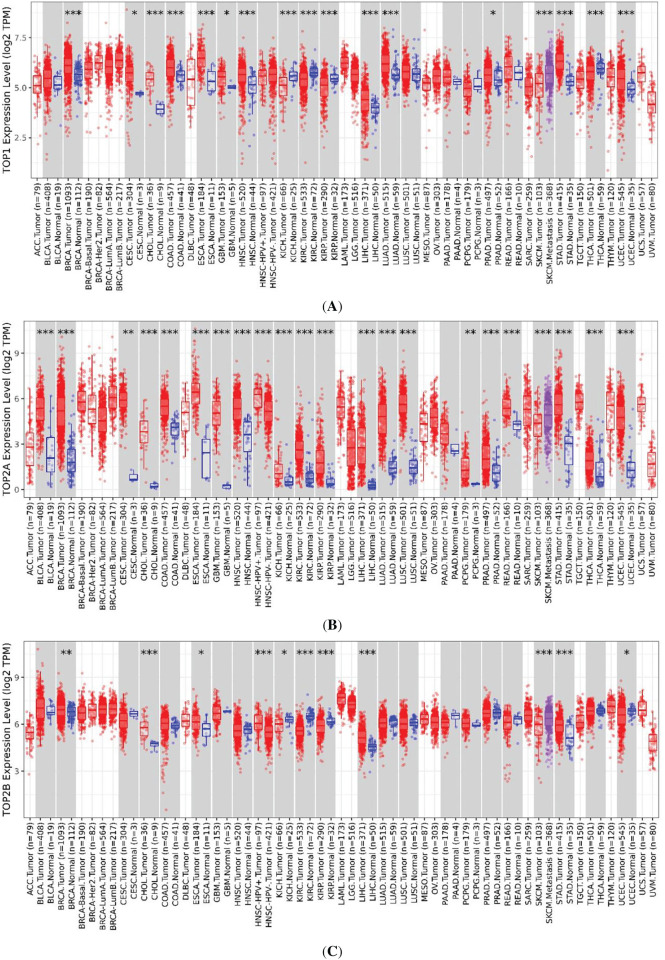

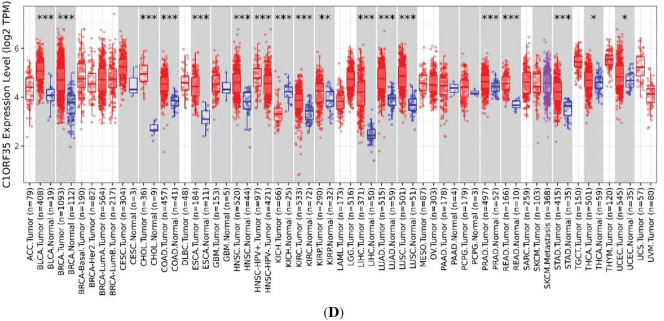
Comparative analysis of (**A**) TOP1, (**B**) TOP2A, (**C**) TOP2B, and (**D**) C1orf35 gene expression in tumor vs. normal tissues across multiple cancer types. The statistical significance is denoted by *, **, and ***, representing *p*-values of <0.05, <0.01, and <0.001, respectively

The expression profile of TOP2B was evaluated across a wide range of tumor and normal tissues using TCGA datasets. As illustrated in Fig. 19, TOP2B expression showed significant dysregulation in several cancer types. Notably, tumors such as bladder urothelial carcinoma (BLCA), colon adenocarcinoma (COAD), head and neck squamous cell carcinoma (HNSC), liver hepatocellular carcinoma (LIHC), and rectum adenocarcinoma (READ) demonstrated significantly elevated TOP2B expression compared to their corresponding normal tissues (*p* < 0.001). In particular, LIHC tumors exhibited markedly higher TOP2B expression, emphasizing its potential role in hepatocarcinogenesis. These findings suggest that aberrant expression of TOP2B may be involved in tumor progression and could serve as a potential biomarker or therapeutic target in multiple cancer types, including hepatocellular carcinoma. Moreover, the differential expression of C1orf35 was analyzed across multiple tumor and normal tissue samples using TCGA datasets. As depicted in Fig. 18, C1orf35 was significantly upregulated in several malignancies, including bladder urothelial carcinoma (BLCA), breast invasive carcinoma (BRCA), colon adenocarcinoma (COAD), head and neck squamous cell carcinoma (HNSC), liver hepatocellular carcinoma (LIHC), lung adenocarcinoma (LUAD), and rectum adenocarcinoma (READ) when compared to their corresponding normal tissues (*p* < 0.001). Notably, LIHC samples exhibited markedly higher C1orf35 expression relative to normal liver tissues, suggesting a potential oncogenic role in hepatocellular carcinoma. These findings highlight the relevance of C1orf35 as a candidate for further functional studies and its potential implication in cancer diagnosis and prognosis. Overall, the consistent overexpression of TOP1, TOP2A, TOP2B, and C1orf35 in tumors compared to normal tissues underscores their relevance in tumor biology and highlights their promise as candidate biomarkers for cancer diagnosis and treatment.

Our comprehensive analysis indicates that the genes TOP1, TOP2A, TOP2B, and C1orf35 are significantly overexpressed in hepatocellular carcinoma (HCC) tissues compared to adjacent non-tumorous liver tissues. This overexpression correlates with advanced tumor stages, as demonstrated by both mRNA expression data and immunohistochemical analyses, which reveal elevated protein levels of these genes in higher-grade HCC samples. Despite the elevated expression levels, the highest alteration frequencies observed were approximately 1.5% for TOP1, 3% for TOP2A, 2% for TOP2B, and 8% for C1orf35. These findings suggest that mechanisms other than gene amplification, such as transcriptional regulation or post-transcriptional modifications, may contribute to their overexpression. Regarding specific mutations, the TOP1 E695K mutation has not been reviewed by the OncoKB (https://www.oncokb.org/) team, and its biological significance remains unknown [54]. Similarly, mutations in TOP2A (including K1256R, K1255E, K1250E, P886S, P881S, H699Q, E76Q, E71Q, and E62K) and frameshift deletions (such as W619*, W614*, and V174Lfs22*) have not been specifically reviewed by OncoKB, leaving their clinical implications unclear [54]. There are currently no FDA-approved or NCCN-compendium listed treatments specifically targeting these TOP2A mutations in HCC patients. For TOP2B, mutations like K1256R, K1255E, K1250E, P886S, P881S, H699Q, E76Q, E71Q, and E62K, along with frameshift deletions such as W619*, W614*, and V174Lfs22*, lack information in OncoKB, and their biological significance is yet to be determined. Additionally, mutations in C1orf35, including G157E, F148Sfs6*, and X82_splice, have not been reviewed by the OncoKB team, and their clinical relevance remains unknown [54]. These findings underscore the need for further research to elucidate the roles of these gene alterations in HCC pathogenesis and their potential as therapeutic targets.

Our analysis of the TIMER database reveals that the genes TOP1, TOP2A, TOP2B, and C1orf35 exhibit significant associations with immune cell infiltration in hepatocellular carcinoma (HCC). Specifically, elevated expression levels of these genes correlate positively with increased infiltration of various immune cells, including CD8^+^ T cells, CD4^+^ T cells, B cells, neutrophils, and macrophages. Notably, TOP1 and TOP2B expressions show strong positive correlations with macrophage and B cell infiltration, suggesting their potential roles in modulating the tumor immune microenvironment. Similarly, TOP2A expression is significantly associated with higher tumor purity and increased infiltration of multiple immune cell types, indicating its involvement in both tumor progression and immune landscape modulation. C1orf35 expression also correlates positively with tumor purity and various immune cell infiltrations, implying its dual role in tumor development and immune interaction. These findings underscore the potential immunomodulatory functions of these genes in HCC and highlight their promise as biomarkers or therapeutic targets in liver cancer.

In summary, our findings highlight the significance of TOP1, TOP2A, TOP2B, and C1orf35 overexpression in HCC pathogenesis and prognosis. The gene’s involvement in vital cellular processes and its potential as a therapeutic target warrant further investigation to elucidate the underlying mechanisms driving its upregulation and to develop targeted treatment strategies for HCC patients. These findings suggest these genes may contribute to shaping the immune landscape of HCC, potentially through pathways that enhance the recruitment or activation of lymphocytes, and thus, it may represent a valuable immunological biomarker or therapeutic target. Furthermore, the integration of phosphoproteomic data with clinical parameters enhances our understanding of HCC heterogeneity and may inform personalized therapeutic strategies.

We acknowledge the absence of *in-vitro* and *in-vivo* validation in this work. To address this, future studies will include cross-validation analyses and testing against external datasets. These steps will strengthen the mechanistic support and improve the biological reliability of our computational results.

## 5 Conclusion

We conducted an extensive phosphoproteomic and proteomic investigation into hepatocellular carcinoma (HCC), unveiling a novel protein signature through integrated analyses and protein-protein interaction (PPI) assessments. Our findings highlight significant dysregulation of TOP1, TOP2A, TOP2B, and C1orf35, indicating distinct structural variations and migratory behaviors between tumor and adjacent non-tumorous tissues. Notably, alterations in phosphorylation sites suggest a critical role in HCC malignancy. These insights offer valuable resources for the scientific community, presenting new therapeutic avenues for HCC treatment. Our study also identified potential drug candidates, highlighting molecules that could be repurposed or developed for HCC therapy. Through survival analysis, we examined multiple intersecting markers, uncovering key prognostic insights into how these markers predict cancer patient outcomes. Significantly, our results indicate that elevated gene expression levels in HCC correlate with poor prognosis, underscoring their potential as prognostic biomarkers. Functional enrichment analysis via KEGG and Gene Ontology (GO) pathways suggests that these genes influence liver cancer progression through diverse targets and pathways. Our study emphasizes the upregulation of TOP1, TOP2A, TOP2B, and C1orf35, identifying them as promising diagnostic and prognostic biomarkers. To our knowledge, we are the first to report these phosphorylation sites, although their functions in HCC remain underexplored. Additionally, C1orf35 has not been previously studied in HCC; understanding its function could provide valuable insights into the molecular mechanisms of liver cancer and potentially identify it as a novel biomarker for diagnosis or a target for therapeutic intervention. The substantial expression alterations of these genes may enhance HCC treatment efficacy, reduce mortality rates, and improve patient stratification accuracy. Our future research will focus on designing, repurposing, and discovering drug molecules targeting TOP1, TOP2A, TOP2B, and C1orf35. Additionally, we aim to characterize previously unrecognized proteins to further elucidate their roles in HCC pathogenesis.

***Limitation and Future Experimental Validations***

We acknowledge the absence of *in-vitro* and *in-vivo* experimental validation in the current study. As part of our future research plan, we propose several experimental approaches, including Western blot confirmation of key phosphorylation sites, targeted kinase inhibition assays, and the development of CRISPR/Cas9 knockout models for TOP1, TOP2A, TOP2B, and C1orf35. These studies will provide mechanistic validation and strengthen the biological relevance of our computational findings.

## Supplementary Materials



## Data Availability

The proteomic and phosphoproteomic datasets analyzed in this study were obtained from the publicly available CPTAC database (Proteomic Data Commons). Moreover, they can also be requested from the corresponding author, [Aktham Mestareehi], upon reasonable request.

## Supplementary Materials

The supplementary material is available online at https://www.techscience.com/doi/10.32604/or.2025.073745/s1.

## Abbreviations

Abbreviation: Full Form
HCC: Hepatocellular carcinoma
AFP: Alpha-fetoprotein
AST: Aspartate aminotransferase
ALB: Albumin
PTT: Partial Thromboplastin Time
GGT: Gamma-Glutamyl Transferase
TB: Total Bilirubin
ALT: Alanine aminotransferase
CPTAC: Clinical Proteomic Tumor Analysis Consortium
TCPA: The Cancer Proteome Atlas
TCGA: The Cancer Genome Atlas
KIRC: Kidney renal clear cell carcinoma
LIHC: Liver hepatocellular carcinoma
STAD: Stomach adenocarcinoma
COAD: Colon adenocarcinoma
CI: Confidence interval
HR: Hazard ratio
BP: Biological process
CC: Cellular component
FDR: False discovery rate
GO: Gene ontology
KEGG: Kyoto Encyclopedia of Genes and Genomes
LC-MS/MS: Liquid chromatography–tandem mass spectrometry
MF: Molecular function
PPI: Protein–protein interaction
RPLC: Reversed-phase liquid chromatography
